# Elucidation of the Structure–Activity Relationship for Cu-Erionite in the Direct Conversion of Methane to Methanol: An Operando XAS Study

**DOI:** 10.1021/jacs.5c03554

**Published:** 2025-06-20

**Authors:** Jie Zhu, Vitaly L. Sushkevich, Amy J. Knorpp, Mark A. Newton, Toru Wakihara, Tatsuya Okubo, Zhendong Liu, Jeroen A. van Bokhoven

**Affiliations:** † Institute for Chemical and Bioengineering, 27219 ETH Zurich, Vladimir-Prelog-Weg 1, Zurich 8093, Switzerland; ‡ Center for Energy and Environmental Sciences, Paul Scherrer Institute, Villigen PSI 5232, Switzerland; § Department of Structure and Dynamics in Catalysis, J. Heyrovsky Institute of Physical Chemistry, Dolejškova 2155/3, Prague 8 182 23, Czech Republic; ∥ Department of Chemical System Engineering, 12442 The University of Tokyo, 7-3-1 Hongo, Bunkyo-ku, Tokyo 113-8656, Japan; ⊥ Institute of Engineering Innovation, The University of Tokyo, 2-11-16 Yayoi, Bunkyo-ku, Tokyo 113-8656, Japan; # State Key Laboratory of Chemical Engineering, Department of Chemical Engineering, Tsinghua University, Haidian, Beijing 100084, China

## Abstract

The partial oxidation of methane to methanol over copper-exchanged zeolites offers a promising avenue for methane valorization. Numerous zeolites have been demonstrated to be active for the selective oxidation of methane, with the methanol yield varying significantly depending on the zeolite framework, Si/Al ratio, and copper loading. Herein, we present a comprehensive study of one of the most active Cu-erionite (Cu-ERI) zeolites with different compositions for the stepwise conversion of methane to methanol, aiming to elucidate the relationship between the methanol yield and the nature of copper species in Cu-ERI zeolites. Operando X-ray absorption spectroscopy (XAS), combined with Fourier-transform infrared spectroscopy (FTIR), allows us to establish a correlation that reveals the dependence of the methanol yield on the reduction rate of copper species. Our findings demonstrate that the Cu/Al ratio plays a crucial role in determining the reducibility of copper species in Cu-ERI zeolites, which in turn governs methanol yield normalized to the copper content. While the Si/Al ratio of the parent zeolite determines the achievable copper loading and the maximal methanol yield, it does not influence the normalized methanol yield. This work suggests that controlling the Cu/Al ratio is essential for maximizing copper efficiency and achieving selective methane partial oxidation. At a fixed optimal Cu/Al ratio, increasing the Al content enhances the total methanol yield by providing more copper exchange sites. The structure–activity relationship of Cu-ERI zeolites in the direct conversion of methane to methanol offers valuable insights into the interplay between the zeolite host and copper species, highlighting the importance of both Cu/Al and Si/Al ratios in designing selective, high-performance materials for this challenging reaction.

## Graphical abstract

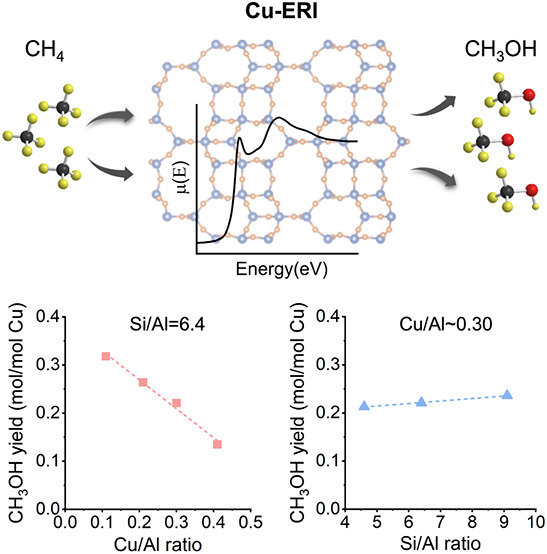

## Introduction

The direct conversion of methane to value-added chemicals is currently an extensively investigated field due to the low cost and enormous potential of methane as a feedstock that continues to be flared on a large scale.^1–6^ Of particular interest is the partial oxidation of methane to methanol, which remains a considerable challenge in the field of hydrocarbon chemistry.^7–12^ Thermodynamically, the primary product of partial oxidation—methanol—is susceptible to further oxidation, which can lead to the formation of undesired overoxidation products such as carbon monoxide (CO) and carbon dioxide (CO_2_).^13–17^ Kinetically, there is competition in the rates of partial oxidation and overoxidation, with the latter consuming more active copper species.^18–20^ Significant efforts have been devoted to optimizing methanol yield through material design and product protection over the past years.^4,9–12,21–33^ One of the most promising approaches involves a cyclic chemical looping process using metal-containing zeolites, which enables high selectivity toward methanol by preventing the further oxidation of methoxy species stabilized within the zeolite framework.^11,24,34–35^

Groothaert and coworkers first demonstrated the possibility of copper-exchanged zeolites selectively converting methane to methanol and identified bis-μ-oxo dicopper species ([Cu_2_(μ-O)_2_]^2+^) as the active sites in Cu-ZSM-5 and Cu-mordenite (Cu-MOR) zeolites.^21^ Following this pioneering work, copper-containing zeolites with various topologies including Cu-MOR, Cu-ZSM-5, Cu-CHA, Cu-MAZ, and Cu-FAU have been extensively investigated for the stepwise conversion of methane to methanol.^24,25,27,36–37,38,39–46^ As a result of these efforts, the methanol yield per cycle continues to increase, and substantial differences have been observed among the copper-containing materials in terms of methanol yield and the structure of active sites.^21,22,36,40,47–50^ Depending on the zeolite topology, material compositions, and reaction conditions (e.g., temperature, pressure, and operation protocol), copper active sites with different structures can be formed.^24,28,36,38,40–42,51–54^ On the basis of various characterization techniques and DFT calculations, copper species with different nuclearities have been proposed as the active sites for C–H bond cleavage.^23,26,30,40,42,44,47,52–61^ Cu-oxo centers comprising dicopper mono-μ-oxo species [Cu_2_O]^2+^,^42,44,59^ peroxo species [Cu_2_O_2_]^2+^,^42^ tricopper species [Cu_3_O_3_]^2+^, and paired monomeric copper species [Cu­(OH)]^+^ have been suggested to be responsible for the activity.^36,30,40,47,57,61–63^ Various types of copper species are expected to exhibit diverse natures/reducibilities and thus lead to different performances in terms of activity and selectivity.

Specifically, the Si/Al ratio and the copper loading (typically represented by the Cu/Al ratio) play important roles in determining the structure and location of the copper active sites. Different copper species are expected to form by balancing the negative charge of the framework Al atoms. The Si/Al ratio, and consequently the proximity of Al atoms, exerts a significant influence on the structure of active sites.^36,51,64–67^ Sushkevich et al. studied the dependence of the type of active sites in Cu-MOR zeolites on the Si/Al ratio, which nominally governs the potential number of Al pairs within the MOR framework channels and cages. From this systematic study, oligomeric copper species (presumably, the [Cu–O–Cu]^2+^ motif) were deduced to dominate in the case of a low Si/Al ratio, whereas monomeric copper species prevail at a high Si/Al ratio, both of which can be functional toward methane partial oxidation but exhibit varied reducibility.^36^ Copper loading is another critical parameter that determines the potential yield achievable in a single cycle. Its influence on the methanol yield, however, varies with the zeolite structure. For instance, a linear trend has been observed in Cu-MOR and Cu-CHA with increasing copper loading;^38,68^ however, for other structures like Cu-MAZ, the behavior is more complex, as the location of copper species can vary depending on both copper loading and the reaction conditions. While copper in the eight-membered rings is potentially active, elevated temperatures and dehydration can cause copper to migrate into the six-membered rings, leading to deactivation. This dynamic behavior results in a distinctly different activity profile compared to other zeolite structures.^37,46^ These findings underscore how methanol yield is strongly influenced by the structural and compositional features of copper-containing zeolites, reflecting the intricate structure–activity relationship.^66,69,70^

Erionite (ERI) zeolite is a small-pore zeolite that has rarely been explored.^71–73^ The structure of ERI zeolite consists of three building units, the cancrinite (*can*) cage, double six-ring (*d6r*), and erionite (*eri*) cage. The *eri* cage features a spacious cavity of approximately 6.3 × 13.0 Å that can be accessed through eight-membered ring openings measuring 3.6 × 5.1 Å, making ERI zeolite suitable for selective catalysis.^74,75^ We recently reported that copper-exchanged ERI zeolite achieved a high methanol yield for the direct oxidation of methane to methanol.^72^ Cu-ERI provides a unique confined environment for copper species, which enables high selectivity toward methanol during the reaction with methane at 300 °C, leading to a methanol yield as high as 147 μmol/g_–zeolite_ with an optimal Cu/Al ratio of 0.30.

Pursuing a comprehensive exploration of the compositional effects on copper species and a deeper understanding of their behavior in Cu-ERI zeolites, we herein present a systematic investigation of Cu-ERI zeolites with varied Cu/Al and Si/Al ratios for the partial oxidation of methane to methanol. By combining in situ Fourier-transform infrared spectroscopy (FTIR) and operando X-ray absorption spectroscopy (XAS), we aim to elucidate the structure–activity relationship of Cu-ERI zeolites. Our findings highlight that the Cu/Al ratio is the key parameter governing the reducibility of copper species, which results in variations in the methanol yield normalized to copper content. This work suggests that adding more copper does not always result in a higher methanol yield. It is crucial to maintain a low copper loading (Cu/Al < 0.3) to achieve selective methane partial oxidation. On the other hand, at a fixed Cu/Al ratio, the Si/Al ratio does not affect the redox properties of copper species, while increasing the Al content can enhance the total methanol yield by providing more copper exchange sites. Additionally, the absolute amount of inactive copper species remains constant for Cu-ERI materials with different Cu/Al ratios but decreases as the Si/Al ratio increases, likely due to the presence of residual K^+^ in the samples.

## Results

### Characteristics of Cu-ERI Zeolites

The synthesis and characterization of Cu-ERI samples are detailed in Supporting Information. ERI zeolites with different Si/Al ratios were prepared using a single organic structure-directing agent, hexamethonium bromide, as the template.^76^ Table 1 summarizes the elemental compositions of Cu-ERI materials with different Si/Al ratios and copper loadings. The samples are designated as “Cu-ERI-*x*(*y*)”, where “*x*” and “*y*” denote the Si/Al and Cu/Al ratios, respectively. For the first series of samples, all Cu-ERI zeolites have a Si/Al ratio of 6.4, with Cu/Al ratios of 0.11, 0.21, 0.30, and 0.41, respectively. The powder XRD patterns (Figure S1) suggest that all four Cu-ERI samples are fully crystalline, with no reflections attributed to other phases, such as copper oxide, being detected. The second series of samples includes three Cu-ERI zeolites with similar Cu/Al ratios (∼0.30) but different Si/Al ratios of 4.6, 6.4, and 9.1. Scanning electron micrographs (Figure S2) indicate the high crystallinity of these samples, whose average particle size decreases from 1.6 to 0.5 μm with increasing Si/Al ratio.

**Table 1:** Elemental Compositions of Cu-ERI Zeolites

	Composition^a^			Cu
Sample	Si/Al	Cu/Al	K/Al	(wt %)
Cu-ERI-6.4(0.11)	6.4	0.11	0.21	1.53
Cu-ERI-6.4(0.21)	6.4	0.21	0.21	2.89
Cu-ERI-6.4(0.30)	6.4	0.30	0.21	4.08
Cu-ERI-6.4(0.41)	6.4	0.41	0.21	5.49
Cu-ERI-4.6(0.29)	4.6	0.29	0.22	5.13
Cu-ERI-9.1(0.30)	9.1	0.30	0.24	3.03

a Determined by ICP measurement.

Infrared spectroscopy was first applied to investigate the nature of the copper species in the Cu-ERI zeolites. Adsorption of nitrogen monoxide (NO) at low temperature allows for the reliable identification of Cu^II^ and Cu^I^ species in copper-containing samples.^36,35,48,69,77,78^ Typically, NO was dosed gradually until complete saturation of the samples (Figure S3). Figure 1a compares the FTIR spectra collected upon NO adsorption over the activated Cu-ERI-6.4­(*y*) zeolites with varied Cu/Al ratios (*y* = 0.11, 0.21, 0.30, and 0.41). The bands observed at 1957, 1945, and 1918 cm^–1^ are assigned to the vibrations of NO adsorbed on Cu^II^ sites. It is generally accepted that the bands at lower vibration frequencies (∼1940 cm^–1^) originate from NO molecules adsorbed on isolated copper sites, likely copper monomers, whereas those at higher vibration frequencies (>1940 cm^–1^) correspond to NO adsorbed on bulk Cu^2+^ ions or copper-oxo sites, such as dimers or trimers.^37,79–82^ The peak centered at 1875 cm^–1^ is ascribed to the vibration of physisorbed NO. The appearance of bands within the range of 1700 ∼ 1850 cm^–1^ is associated with NO adsorbed on Cu^I^ sites. Specifically, the band at 1787 cm^–1^ is due to the formation of Cu^I^NO mononitrosyl, while those at 1825 and 1713 cm^–1^ correspond to Cu^I^(NO)_2_ dinitrosyl species.^36,35,80,81,82^ The band at 1677 cm^–1^ ( $\vartheta_{as}$ ) along with that at 2025 cm^–1^ ( $\vartheta_{s}$ ) are presumed to be associated with the vibration of [N_2_O_2_]^+^ species.^83,80^ The presence of multiple bands at the same frequencies indicates that the same types of copper species were formed in all four Cu-ERI samples, comprising a mixture of both monomeric copper species and copper-oxo sites with varying numbers of copper atoms (Figure 1a). However, the fraction of each type of copper site varied depending on the Cu/Al ratio. The intensity of the band at 1918 cm^–1^ increases proportionally with the Cu/Al ratio (Figure S4), indicating that more copper monomers are formed in Cu-ERI zeolites with higher copper loading. Meanwhile, the intensity of the band at 1957 cm^–1^ is maximal for Cu-ERI-6.4(0.30), suggesting that this sample contains the highest fraction of copper-oxo sites and/or bulk Cu^2+^.^79^ This observation also points to the possible presence of species invisible to NO FTIR, such as copper clusters and larger particles.^35^ Figure S5 compares the FTIR spectra of adsorbed NO for Cu-ERI-6.4(0.30) after activation in oxygen and reaction with methane, respectively. Upon reaction with methane, all types of Cu^II^ species are consumed, as evidenced by the decrease in the intensities of the bands within 1900 ∼ 2000 cm^–1^, accompanied by the formation of Cu^I^ species. The spectral change indicates that selective consumption of a single type of active site was not observed; instead, all Cu^II^ species comprising different nuclearities exhibit high reactivity toward methane.

**Figure 1:**
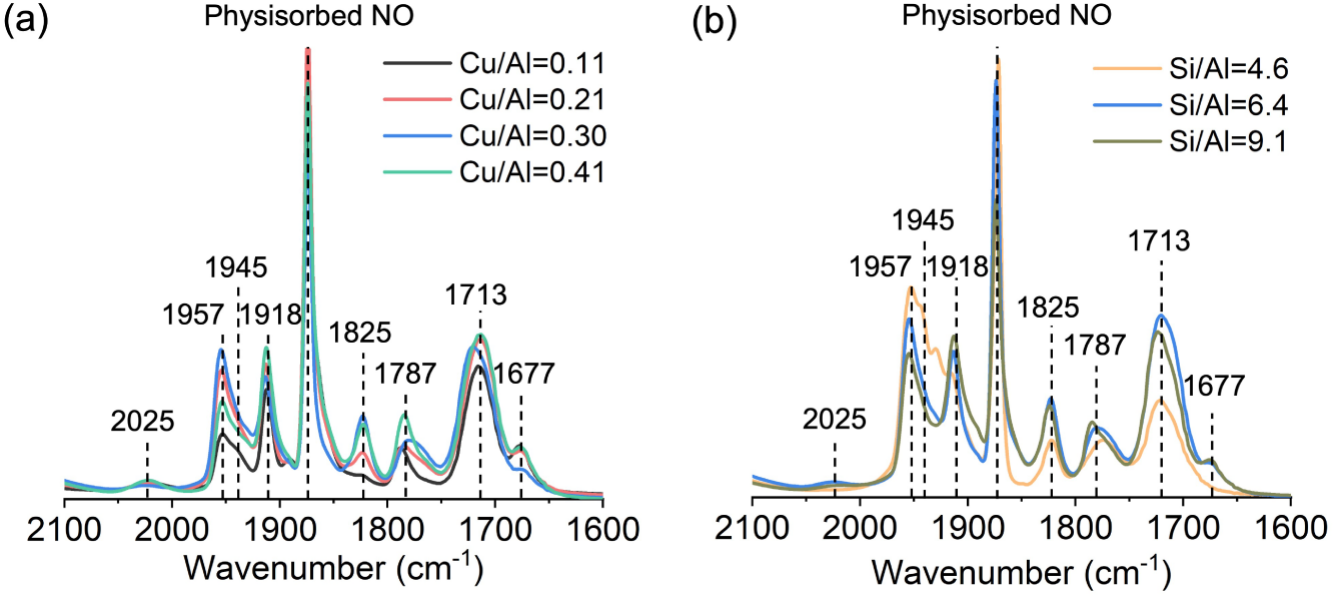
In situ FTIR spectra of nitrogen monoxide over (a) Cu-ERI-6.4­(*y*) zeolites with different Cu/Al ratios; (b) Cu-ERI-*x*(0.30) zeolites different Si/Al ratios after activation in 300 Torr of oxygen at 400 °C for 1 h. *x* and *y* denote the Si/Al and Cu/Al ratios, respectively.

The Si/Al ratio of Cu-ERI zeolite, on the other hand, was found not to alter the types of copper species, as the spectra of adsorbed NO in all three activated Cu-ERI-*x*(0.30) samples appeared at the same frequency (Figure 1b). The intensity of the band at 1918 cm^–1^ increased with the Si/Al ratio, indicating a predominant formation of monomeric copper species in Cu-ERI-9.1(0.30). In contrast, Cu-ERI-4.6(0.30) exhibited the highest intensity of the high-frequency NO band (>1940 cm^–1^), likely due to the higher fraction of Al pairs associated with its low Si/Al ratio, which leads to the formation of more copper-oxo and/or bulk Cu^2+^ species. A drastic decline in the intensity of the bands at 1825 and 1713 cm^–1^ indicates less formation of Cu^I^ species in Cu-ERI-4.6(0.30) compared to the other two samples, suggesting that the copper-oxo species are more resistant toward reduction under vacuum.

Figure 2a,b displays the ex situ Cu K-edge *k* ^3^-weighted EXAFS spectra collected over oxygen-activated Cu-ERI samples, sealed in thin-walled capillaries, at low temperature. No pronounced differences in the *k*-space EXAFS spectra were observed among Cu-ERI samples with different Cu/Al and Si/Al ratios. The presence of shoulders at 5, 8, and 12 Å^–1^ resembled the features observed in Cu-FAU and Cu-BEA,^54^ which indicates the formation of different types of copper-oxo species with a number of copper atoms greater than 2. After Fourier transform (FT), two main regions were observed at phase-uncorrected radial distances of approximately 1.5 and 2.3 Å (Figure 2c,d). The first peak is generally attributed to scattering from oxygen atoms surrounding the copper atoms, and the second peak comprises contributions from the framework aluminum/silicon atoms and extraframework copper species.^84,38,35,54,85^ Similar to the *k*-space spectra, the FT EXAFS spectra of Cu-ERI zeolites do not exhibit substantial changes upon varying the Cu/Al and Si/Al ratios. The peak appearing at 2.8 Å suggests the presence of a second copper atom in copper-oxo species, as demonstrated in previous studies, suggesting the formation of small copper-oxo aggregates.^54^

**Figure 2:**
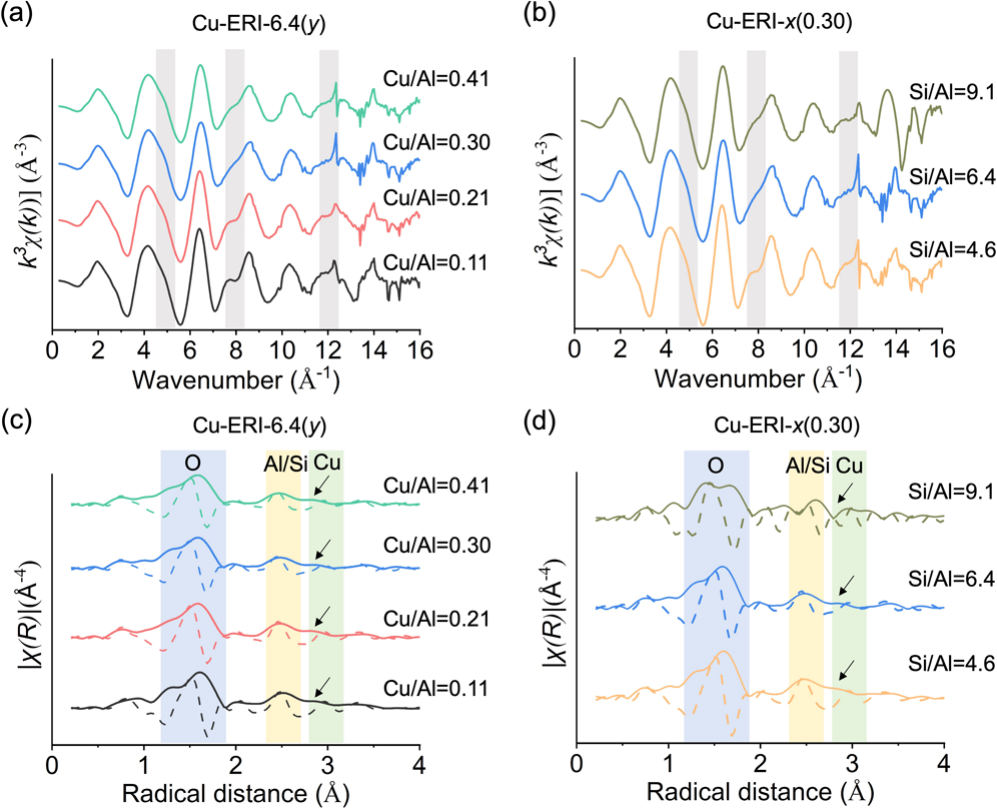
*k* ^3^-weighted χ­(k) data collected over (a) activated Cu-ERI-6.4­(*y*) and (b) activated Cu-ERI-*x*(0.30) zeolites acquired at −140 °C; phase-uncorrected FT EXAFS spectra obtained from *k* ^3^-weighted χ­(k) data of (c) activated Cu-ERI-6.4­(*y*) and (d) activated Cu-ERI-*x*(0.30) zeolites in the range of 3–16 Å^–1^. The samples were activated at 450 °C in flowing oxygen for 1 h. Gray sectors indicate the shoulders assigned to the second copper-oxo species. Colored sectors denote the main contributors to the scattering in the selected regions: oxygen (blue), aluminum, silicon, or copper (yellow), and exclusively copper (green). Solid and dotted lines correspond to the magnitude and real parts of the Fourier transform, respectively. The arrows point to the presence of additional contributions in the second coordination shell.

### Tracking Fingerprints of the Copper Species by Operando XAS

To further analyze the change in the oxidation state and coordination environment of copper species during the chemical looping process, operando XAS measurements were carried out on Cu-ERI zeolites with different compositions during the conversion of methane to methanol. Figure S6 illustrates the schematic of the in situ XAS experiment, during which Cu K-edge X-ray absorption near-edge structure (XANES) spectra were recorded throughout the entire chemical looping process, and EXAFS spectra were collected after each reaction step. Figures 3a and S7a,c,e depict the normalized Cu K-edge XANES spectra of the Cu-ERI-6.4­(*y*) zeolites with different Cu/Al ratios after being heated to 450 °C and subsequently activated in oxygen for 1 h. The appearance of the weak pre-edge feature at 8977 eV and the rising edge feature at 8987 eV indicates the presence of Cu^II^ species, which correspond to the 1s→3d transition and the 1s→4p transition of Cu^II^, respectively.^24,30,48,55,86,87^ The decline in intensity of the white line at 8997 eV, along with increasing temperature, is indicative of the removal of water molecules coordinated to the copper species.^24,30,66^ These observations are in line with previous literature demonstrating that the activation of copper zeolites in oxygen at elevated temperatures leads to the conversion of octahedrally coordinated Cu^II^ aqua-complexes to framework-coordinated Cu^II^ species.^84,44,52^ XANES spectra collected after the main steps of the reaction protocol are all presented in Figures 3b and S7b,d,f. The data suggest that the oxidation state of the copper species remains unchanged after switching the reaction system from oxygen to helium and that the autoreduction of Cu^II^ species in Cu-ERI zeolites is negligible at 300 °C.^33,38,39^ Upon reaction with 1 bar of methane, a significant fraction of Cu^I^ species was formed, as evidenced by the appearance of an absorption feature at 8983 eV, attributed to the 1s→4p transition of Cu^I^.^24,30,87^ In addition, a new peak at 8989 eV was observed, which is also assigned to Cu^I^ species.^35,88^ The shape of the spectrum changed markedly after water-assisted methanol extraction, yielding an intensity increase of the feature at 8983 eV together with a decline in intensity of the peak at 8989 eV. We assume that this shape change in the XANES spectrum is due to the variation of the copper local environment upon the removal of methoxy species.

**Figure 3:**
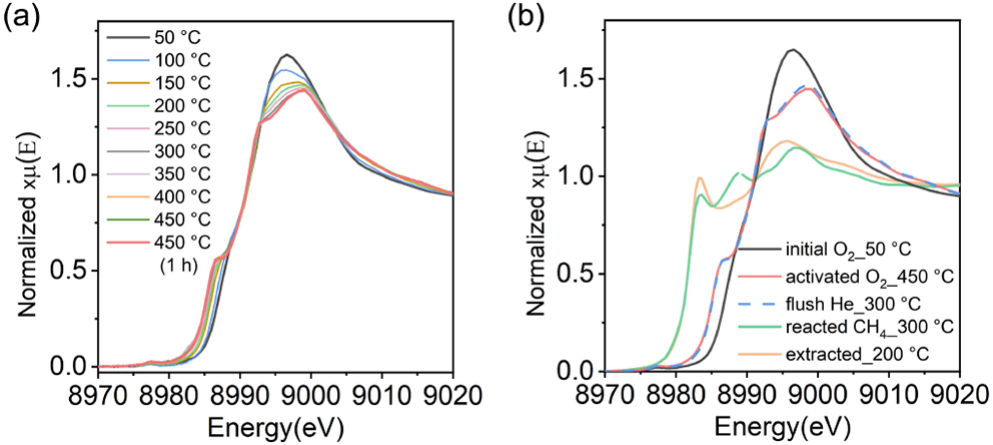
(a) Cu K-edge XANES of Cu-ERI-6.4(0.11) during activation in oxygen for 1 h; (b) Cu K-edge XANES of Cu-ERI-6.4(0.11) collected after each step in the conversion of methane to methanol: before activation at 50 °C, activation in oxygen at 450 °C, helium flush after cooling to 300 °C, reaction with 1 bar of methane at 300 °C, and water-assisted extraction of methanol at 200 °C.

To understand the relationship between methanol yield and the composition of copper species in Cu-ERI materials, we subsequently compared the in situ XANES spectra collected on the four Cu-ERI-6.4­(*y*) samples (*y* = 0.11, 0.21, 0.30, and 0.41) after activation in oxygen and reaction with methane. No significant difference was observed with respect to the oxidation state of the copper species across the Cu-ERI-6.4 samples with varying Cu/Al ratios (Figure 4a). This observation was further confirmed by the in situ *k* ^2^-weighted, phase-uncorrected FT EXAFS spectra (Figure S8). Figure 4b shows the magnitude part of the FT-EXAFS spectra in the R-space of the oxygen-activated Cu-ERI-6.4­(*y*) materials. The first-shell peak centered at a radial distance of about 1.4 Å (phase-uncorrected) is associated with Cu–O single scattering paths.^30,38,44,54,55,66,89^ The intensities of this peak are comparable across the four samples, suggesting that on average, the coordination environment of the copper species is similar after activation in oxygen. The well-defined spectral features above 2 Å are attributed to contributions from framework Al or Si atoms, or Cu–Cu scattering paths.^84,30,54,90–92^ It was found that the intensity of the second peak decreased with the Cu/Al ratio, possibly indicating that the interaction between copper species and the framework Al/Si weakens with increasing copper loading. To determine the coordination environment of the copper species in the Cu-ERI-6.4­(*y*) samples, EXAFS fitting analysis (Figure S9–S12) was performed, and the fitted parameters are summarized in Table 2. The fitting results demonstrate that the coordination number (CN) for copper surrounded by oxygen atoms in the activated samples range from 3.7 to 4.0, which is consistent with previous reports showing that four oxygen atoms are present in a square planar configuration.^84,38,44,54,89^ The fitting of the second shell is significantly more challenging, as it generally includes contributions from framework Si and Al atoms, as well as extraframework Cu atoms.^84,54,91^ Moreover, the high temperature leads to the higher Debye–Waller parameters, causing the features within the spectra to broaden and become less distinct, which makes fitting the data more ambiguous.

**Figure 4:**
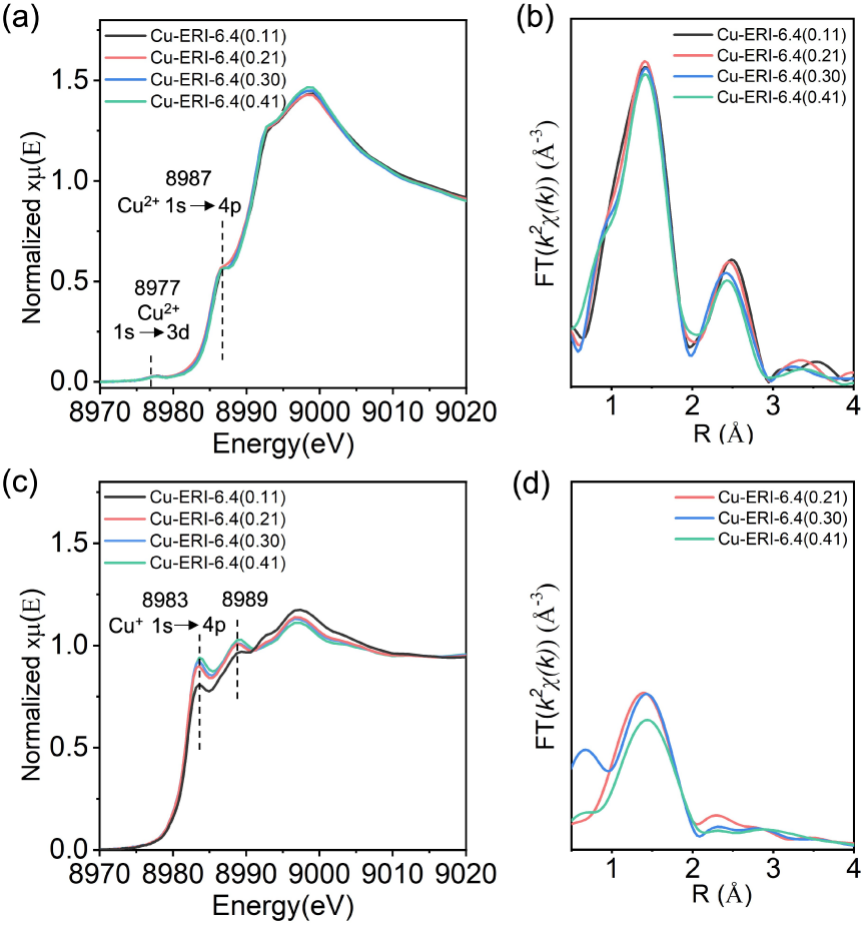
(a) Cu K-edge XANES spectra; (b) magnitude part of the FT-EXAFS spectra of Cu-ERI-6.4­(*y*) zeolites with different Cu/Al ratios after activation in oxygen at 450 °C for 1 h; (c) Cu K-edge XANES spectra; (d) magnitude part of the FT-EXAFS spectra of Cu-ERI-6.4­(*y*) zeolites with different Cu/Al ratios after reaction with 1 bar of methane at 300 °C for 30 min.

**Table 2:** EXAFS Fitting Results of Cu-ERI-6.4­(*y*) Zeolites with Different Cu/Al Ratios after Activation in Oxygen at 450 °C for 1 h^a^

Sample	Path	CN	Distance
Cu-ERI-6.4(0.11)	Cu–O_ss_	4.0 ± 0.2	1.94 ± 0.019
Cu-ERI-6.4(0.21)	Cu–O_ss_	3.9 ± 0.1	1.94 ± 0.017
Cu-ERI-6.4(0.30)	Cu–O_ss_	3.7 ± 0.2	1.94 ± 0.021
Cu-ERI-6.4(0.41)	Cu–O_ss_	3.9 ± 0.1	1.94 ± 0.016

a The fit was performed in the R range of 1.0–3.2 Å while employing the *k*-range of 2.4–10 Å^–1^.

Upon reaction with 1 bar of methane at 300 °C, the absorption features attributed to Cu^I^ species were formed, and their intensity gradually developed with the Cu/Al ratio (Figure 4c). As shown in the FT-EXAFS spectra (Figure 4d), the intensity of the first shell decreased significantly after reacting with methane, suggesting a decrement in the number of oxygen atoms coordinated to copper. The fitted EXAFS CN for copper ranged from 1.3 to 1.7, consistent with the presence of Cu^I^ species, which typically possess a first shell CN of 2 (Figures S13–S16 and Table 3).^84,54^ In addition, an apparent decrease in the intensity of the second shell was observed, which might be associated with the disappearance of Cu–Cu scattering paths in the reacted copper species due to structural disorder in Cu^I^ species.^93^ Similar features have been observed in previous studies and were attributed to the consumption of dimeric copper sites during the reaction.^59,94^

**Table 3:** EXAFS Fitting Results of Cu-ERI-6.4­(*y*) Zeolites with Different Cu/Al Ratios after Reaction with Methane at 300 °C for 30 min^a^

Sample	Path	CN	Distance
Cu-ERI-6.4(0.21)	Cu–O_ss_	1.7 ± 0.1	1.92 ± 0.025
Cu-ERI-6.4(0.30)	Cu–O_ss_	1.6 ± 0.2	1.93 ± 0.043
Cu-ERI-6.4(0.41)	Cu–O_ss_	1.3 ± 0.1	1.94 ± 0.033

a The fit was performed in the R range of 1.0–3.2 Å while employing the *k*-range of 2.4–10 Å^–1^.

Variation of the Si/Al ratio did not result in a detectable change in the XANES spectra collected for the three Cu-ERI-*x*(0.30) samples after activation in a flow of oxygen (Figure 5a), suggesting the dominant presence of Cu^II^ species in all of the samples. This was further confirmed by the *k* ^2^-weighted FT-EXAFS spectra (Figure S17). The fitting of the FT-EXAFS spectra (Figures S11, S18, S19 and Table S1) reveals that the CN of the Cu^II^ species in the first shell is around four. Nonetheless, the sample with the lower Si/Al ratio exhibits a slightly higher CN, which is similar to what have been reported in previous literature.^30,38^ The difference in the intensity of the XAS features (Figure 5b) is likely associated with the heterogeneity of the Cu^II^ species, in line with FTIR of adsorbed NO.^38^ No significant differences were observed in the XANES and FT-EXAFS spectra collected after the reaction with methane (Figures 5c,d and S20), despite the absolute copper loading varying significantly among the three Cu-ERI-x(0.30) samples. The fitted CN of Cu–O for all three samples (Figures S15, S21, S22 and Table S2) is around 1.6, further confirming the similarity in the coordination environment of the copper species across the three Cu-ERI-x(0.30) samples, regardless of the Si/Al ratio.

**Figure 5:**
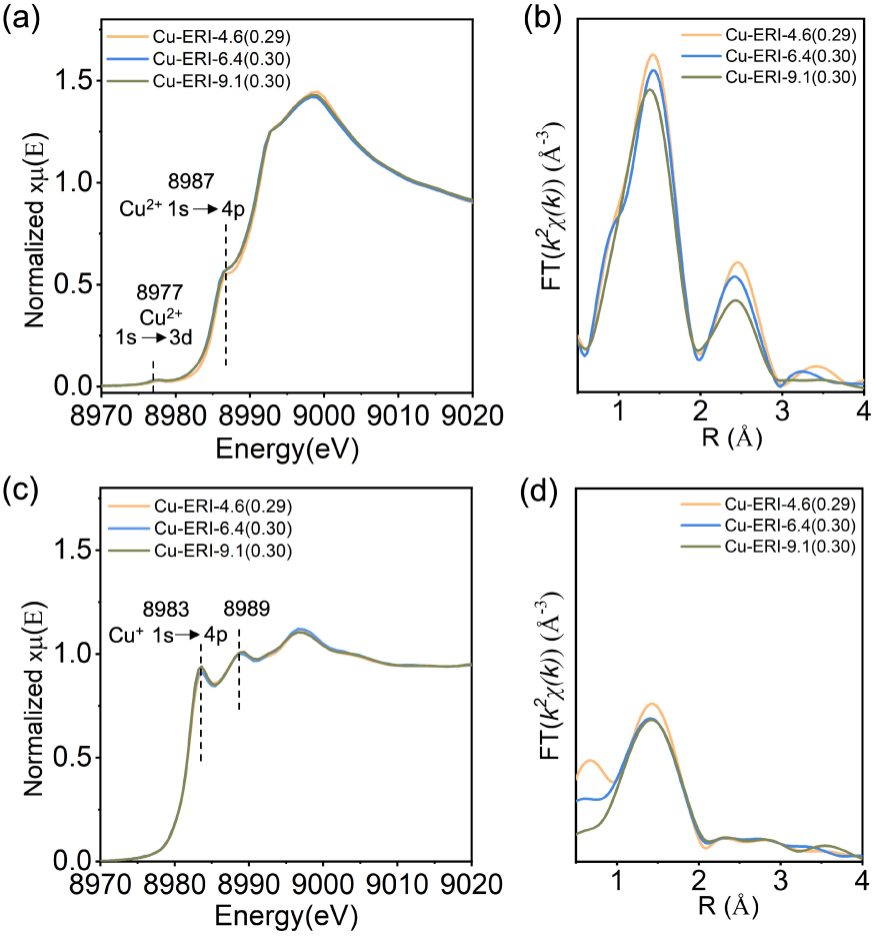
(a) Cu K-edge XANES spectra; (b) magnitude part of the FT-EXAFS spectra of Cu-ERI-*x*(0.30) zeolites with different Si/Al ratios after activation in oxygen at 450 °C for 1 h; (c) Cu K-edge XANES spectra; (d) magnitude part of the FT-EXAFS spectra of Cu-ERI-*x*(0.30) zeolites with different Si/Al ratios after reaction with 1 bar of methane at 300 °C for 30 min.

Linear combination fitting (LCF) analysis was further applied to obtain quantitative insights into copper speciation in the Cu-ERI materials after reaction with methane. Figure S23 shows the time-resolved Cu K-edge XANES spectra acquired during the reaction with 1 bar of methane at 300 °C. Upon contact with methane, Cu^II^ species were gradually reduced to form Cu^I^, as indicated by the development of a rising-edge peak around 8983 eV. The spectra collected after activation in oxygen and the one recorded after reacting with methane at 600 °C were employed as standards for Cu^II^ and Cu^I^, respectively.^66,95,96^ Figure 6a depicts the time-resolved evolution of Cu^II^ and Cu^I^ species during the reaction with 1 bar of methane over Cu-ERI-6.4­(*y*) zeolites. The reduction rate of Cu^II^ to Cu^I^ species is facilitated as the Cu/Al ratio increases. Under the given reaction conditions, it took 1500, 550, and 275 s to convert half the amount of Cu^II^ species in Cu-ERI zeolites with Cu/Al ratios of 0.11, 0.21, and 0.30, respectively. The logarithmic plots for the fraction of Cu^II^ species reveal two distinct kinetic regions with linear behavior (Figure S24), suggesting the presence of at least two different copper sites with varied reducibility, and the relative proportion of these copper sites changes with the Cu/Al ratio.

**Figure 6:**
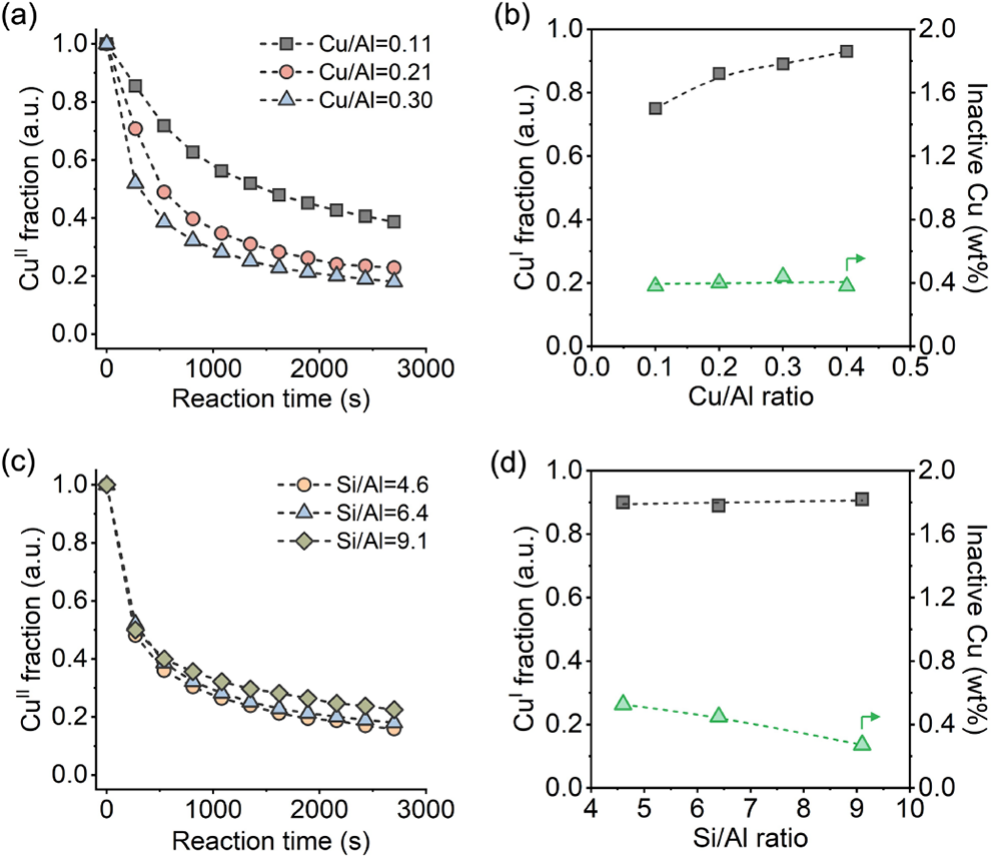
LCF of time-resolved XANES data obtained over (a) Cu-ERI-6.4­(*y*) and (c) Cu-ERI-*x*(0.30) zeolites during the reaction with 1 bar of methane at 300 °C; The fractions of reduced Cu^I^ and inactive copper species in (b) Cu-ERI-6.4­(*y*) and (d) Cu-ERI-*x*(0.30) zeolites after the reaction with 15 bar of methane at 300 °C for 30 min. The amount of unreducible copper Cu wt % is calculated based on the mass of Cu-ERI zeolites.

The fraction of Cu^I^ further increased when a higher methane pressure was applied, as indicated by the enhancement of the feature at 8983 eV in the XANES spectra collected on Cu-ERI-6.4­(*y*) zeolites (Figure S25). Figure S26 shows the Cu^I^ fraction as a function of methane pressure across the four samples, confirming that most of the Cu^II^ species in all samples can be reduced to Cu^I^ at 1 bar of methane, although 15 bar is required to achieve the maximum conversion of Cu^II^. Higher methane pressure leads to a greater involvement of the activated copper sites.^10,38^ The fraction of reduced Cu^I^ in Cu-ERI-6.4(0.11) increases from 65% to 76% when methane pressure is raised from 1 to 15 bar. With increasing Cu/Al ratio, the influence of methane pressure on the reduction of Cu^II^ species becomes less significant. LCF results provide evidence that the fraction of Cu^I^ species formed upon reaction with 15 bar of methane is 75%, 86%, 89%, and 93% for the four Cu-ERI samples with Cu/Al ratios of 0.11, 0.21, 0.30, and 0.41, respectively (Figure 6b). The amount of reducible Cu^II^ species in the Cu-ERI zeolites was substantially higher than that in other types of zeolites.^84,34,38,44,48^ Surprisingly, we found that the four Cu-ERI-6.4­(*y*) samples contained the same amount of irreducible copper species, which was calculated to be approximately 0.4 wt % based on the total mass of the materials (Figure 6b). This fraction of copper species was inactive in the methane oxidation process. It has been reported that Cu-ERI zeolite always contains a small fraction of potassium, which is located inside the *can* cage and cannot be removed from the zeolite framework by ion exchange.^73,76^ We hypothesize that the irreducible copper species might be correlated with the remaining potassium cations in the *can* cage, sitting in the *d6r* along the long cascade, which renders them highly redox-inert and/or inaccessible to methane.^73^

The reduction rate of Cu^II^ to Cu^I^ in the Cu-ERI-*x*(0.30) zeolites, however, was hardly affected by the Si/Al ratio (Figure 6c). This observation aligns with the TPR-CH_4_ results (Figure S27), suggesting that the three samples exhibited comparable reducibility, given the same Cu/Al ratio. Quantitative analysis of Cu^I^ species indicates that a large fraction (90%) of Cu^II^ was reduced to Cu^I^ in the three Cu-ERI-*x*(0.30) zeolites after reacting with methane at 15 bar (Figure 6d), while the sample with a higher Si/Al ratio contained a smaller amount of inactive copper species. As previously discussed, this portion of inactive copper species is likely associated with the remaining potassium species, the quantity of which is determined by the Si/Al ratio of the sample. In summary, these results reveal that the redox properties of the copper species in Cu-ERI zeolites are largely governed by the Cu/Al ratio, which provides valuable guidance for preparing materials for selective methane oxidation.

Finally, methanol extraction was conducted using wet helium flow at 200 °C. Unlike observations in previous studies,^38,97,98^ the fraction of Cu^I^ (8983 eV) in the Cu-ERI zeolites further increased after steam extraction (Figure S28), indicating that Cu^I^ species in Cu-ERI are highly stable in steam. In contrast, previous research on other types of zeolites, such as Cu-MOR or Cu-CHA, has shown that a portion of Cu^I^ species could be reoxidized to Cu^II^ during methanol extraction.^38,48^ For Cu-ERI, however, the Cu^I^ species remained unchanged at 200 °C under steam, indicating an extremely strong interaction between the copper species and the ERI zeolite framework. Meanwhile, the absorption feature at 8989 eV, which is attributed to Cu^I^ species with a different structure,^35^ completely disappeared upon extraction, suggesting that this type of Cu^I^ species may be unstable in the presence of steam. This observation again confirms the presence of two distinct Cu^II^ species in Cu-ERI, which are believed to be associated with the different microenvironments of copper species. After the completion of the first cycle, Cu-ERI-6.4(0.30) was purged with helium and subjected to continuous multicycle testing. As shown in Figure S29, the XANES spectra collected after each step overlapped across three consecutive cycles, indicating that all Cu^I^ species in the Cu-ERI zeolites can be fully reoxidized in oxygen. LCF analysis reveals that the fraction of Cu^I^ formed during the reaction with methane at 300 °C in each cycle remains consistent, demonstrating the good cyclability of the Cu-ERI zeolite.

### Composition–Activity Relationship of Cu-ERI Zeolites

The activity of Cu-ERI zeolites was evaluated by using the direct conversion of methane to methanol via chemical looping. The samples were first activated in oxygen at 450 °C, followed by reaction with 30 bar of methane at 300 °C, and then, methanol was extracted with water at room temperature. As depicted in Figure 7a, the correlation between the total methanol yield achieved over Cu-ERI-6.4­(*y*) zeolites and the Cu/Al ratio exhibits a “volcano” shape, with Cu-ERI-6.4(0.30) yielding the highest methanol yield. When normalized by copper content, increasing the Cu/Al ratio from 0.11 to 0.41 leads to a decrease in the normalized yield, which dropped from 0.318 to 0.135 mol-MeOH/mol-Cu (Figure 7b). This observation exhibits a reverse trend with the fraction of Cu^I^ species obtained over the four Cu-ERI-6.4 samples (Figure 6b), suggesting that only a portion of the reactive copper species participated in the selective oxidation of methane to methanol under the given conditions. The different trends observed in Figure 7a,b highlight the interplay between the total copper content and per-site selectivity. As the Cu/Al ratio increases, the additional copper species become less selective, leading to a decline in per-site efficiency. When the normalized methanol yield is calculated based on the fraction of active copper species (defined as those reducible upon reaction with methane) in each Cu-ERI-6.4­(*y*) sample, rather than the total copper content, a more accurate estimation of the nuclearity of the copper sites can be expected. The methanol yield normalized by the active copper species decreased with the Cu/Al ratio, and the maximum copper efficiency (0.42 mol-MeOH/mol-Cu) was achieved on the sample having the lowest copper loading, which requires an average of two copper atoms to convert one methane molecule.

**Figure 7:**
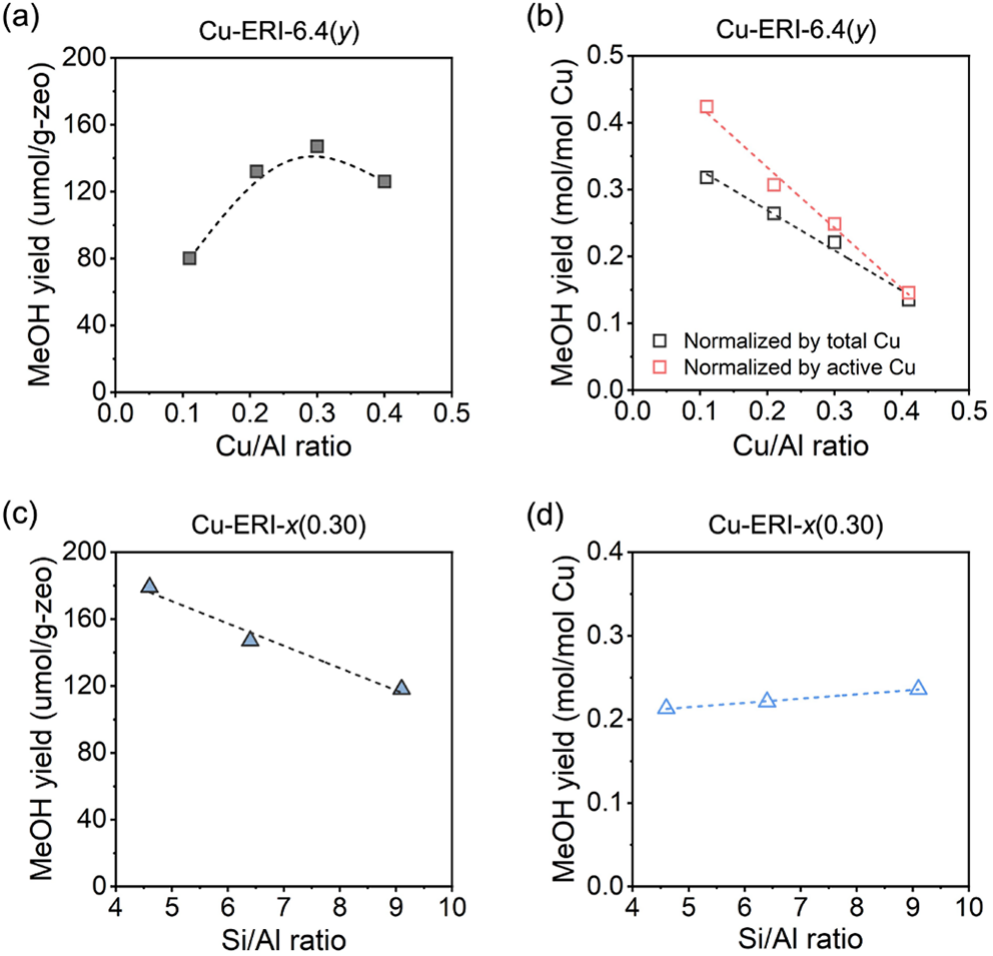
Methanol yield normalized by (a) the mass of zeolite and (b) the copper content over Cu-ERI-6.4­(*y*) with different Si/Al ratios. Methanol yield normalized by (c) the mass of zeolite and (d) the copper content over Cu-ERI-*x*(0.30) zeolites with different Si/Al ratios. Reaction condition: 300 °C under 30 bar of methane after three extractions. *x* and *y* denote the Si/Al and Cu/Al ratios, respectively. Active copper species are defined as Cu^II^ species that are reducible upon reaction with methane.

For the three Cu-ERI-*x*(0.30) samples with different Si/Al ratios, not surprisingly, the total methanol yield increased with decreasing Si/Al ratio (Figure 7c), as more copper could be loaded into the sample with a lower Si/Al ratio. Cu-ERI-4.6(0.30) exhibited the highest methanol yield, with a methanol yield of 179 μmol/g_–zeolite_ achieved after three extractions, representing the highest methanol yield reported for small-pore zeolites to date.^25,27,43,52,72,99^ It was found that the Si/Al ratio appears to have no significant influence on the normalized yield obtained over Cu-ERI zeolites, and the three samples with the same Cu/Al ratio of 0.30 gave an equivalent normalized methanol yield of 0.22 ± 0.01 mol-MeOH/mol-Cu (Figure 7d), unlike other zeolite topologies (Table S3).^19,21,28–84,33,38,40,47,51,70,100^ Our results suggest that in the case of Cu-ERI zeolite, the Si/Al ratio does not markedly impact methanol yield. Instead, the Cu/Al ratio of the Cu-ERI zeolites plays a dominant role in determining the copper-normalized activity.

## Discussion

The investigation of Cu-ERI zeolites with varying Cu/Al and Si/Al ratios provides important insight into the structure–activity relationship of Cu-ERI in the conversion of methane to methanol, which highlights the key parameters toward material design for this complex reaction. The analysis of the FTIR data reveals the presence of similar copper sites across all samples, suggesting that neither the Cu/Al ratio nor the Si/Al ratio influences the types of copper species present, which include both monomeric and copper-oxo sites with higher nuclearity. However, differences in the band intensity indicate that the Cu/Al ratios in the materials affect the relative fraction of each type of copper species (Figure 1). In turn, as demonstrated by FTIR and XAS spectroscopy, the variations in Cu/Al ratios significantly affect the overall redox properties of the samples.

The reducibility of copper species in Cu-ERI, which has a key impact on the normalized methanol yield,^84,38,35,66^ is largely determined by the Cu/Al ratio. When copper species are introduced to H-ERI zeolite, depending on the local distribution of Al in the parent framework, Z_2_Cu^II^ and Z­[Cu^II^OH] species (Z denotes charged zeolite framework), which are associated with two adjacent framework Al atoms and only one Al atom in proximity, respectively, are expected to form at different exchange sites.^65,69,101^ It is generally accepted that bare Z_2_Cu^II^ is redox-inert, while Z­[Cu^II^OH] exhibits high reducibility due to its weaker interaction with the zeolite lattice and the presence of an extraframework oxygen atom.^62,66,69,91^ As the copper loading increases, copper-oxo sites with various nuclearities start to form. The Al atoms can therefore be considered as “anchors” to retain the copper species. The interaction gets weaker when more copper species are loaded into the Cu-ERI zeolites with a fixed Si/Al ratio, resulting in a higher fraction of copper species that are more easily reduced, as revealed by the LCF fitting of XANES spectra collected upon the reaction with methane (Figure 6a). While a substantial proportion of Cu^II^ species (75 ∼ 93%) in Cu-ERI zeolites can be reduced upon reacting with methane, increasing the copper content does not necessarily lead to higher methanol yield. In fact, the total methanol yields achieved by Cu-ERI with different Cu/Al ratios (Figure 7a) exhibit the same trend as the amount of copper-oxo species present in the samples (Figure S4), pointing to copper-oxo species being the most active sites for methane conversion. A two-electron redox mechanism has been proposed for the oxidation of methane to methanol, which proceeds via the reduction of Cu^II^ to Cu^I^ species.^18,19,34^ In contrast, the formation of overoxidized products such as CO and CO_2_ requires six and eight electrons, respectively. When calculating the theoretical methanol selectivity based on the number of electrons required to form different products across the four Cu-ERI samples with varying Cu/Al ratios, it was observed that a higher Cu/Al ratio resulted in decreased methanol selectivity (Figure 8a). Notably, for the copper-exchanged ERI with the highest copper content, Cu-ERI-6.4(0.41), the decline in the intensity of the band at 1957 cm^–1^ suggests the possible formation of large copper clusters, which may contribute to the unselective oxidation of methane. This also aligns with the ex situ EXAFS spectra, suggesting the presence of various copper-oxo species containing more than two copper atoms. On the other hand, Cu-ERI zeolites with different Si/Al ratios exhibited comparable reducibility when the Cu/Al ratio was kept constant (Figure 6c). Despite the differences in absolute Al content, the fraction of Al species charge balanced by copper species was nearly identical among the three Cu-ERI samples with varied Si/Al ratios, resulting in similar interactions with the zeolite framework. The Si/Al ratio of Cu-ERI zeolites appears to have a minimal impact on the normalized methanol yield. Varying the Si/Al ratio in ERI zeolite maintained overall copper efficiency, as plotted in Figure 8a, where the three Cu-ERI-*x*(0.30) achieved comparable methanol selectivity. Collectively, these insights indicate that low copper loading (Cu/Al < 0.3) is critical to direct selective methane partial oxidation, and high Al content is beneficial to improve total methanol yield at a given Cu/Al ratio.

**Figure 8:**
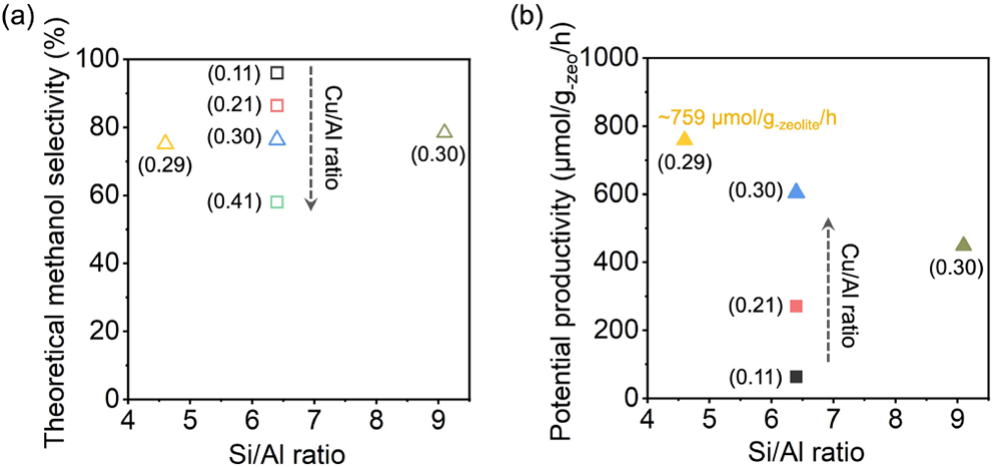
(a) Theoretical methanol selectivity across Cu-ERI zeolites with varying Cu/Al and Si/Al ratios. Theoretical methanol selectivity is calculated as the moles of methanol divided by the total moles of methanol and other products that require six electrons to form; (b) potential productivities that can be derived from Cu-ERI zeolites with different compositions. These productivity values are calculated based on the time required to reduce 50% of Cu^II^ in each sample, an estimated desorption time of 400 s, and a reoxidation time equal to the reduction time.^45^ The numbers in parentheses represent the Cu/Al ratio.

Figure 8b shows the potential productivities that can be derived from Cu-ERI zeolites with different compositions. These productivity values are calculated based on the time required to reduce 50% of Cu^II^ in each sample, with the reduction time determined from the current work (Figures 6a,c), and the estimated desorption and reoxidation times—both of which are based on observations from previous work—set at 400 s and equal to the reduction time, respectively.^45^ The potential productivity increases with both copper loading in the three Cu-ERI-6.4­(*y*) samples (*y* = 0.11, 0.21, and 0.30) and the Al content in ERI zeolites (Figure 8b). In particular, the highest productivity value of ∼759 μmol/g_–zeolite_/h was calculated for Cu-ERI-4.6(0.30), which is approximately one-quarter of the productivity of 3000 μmol/g_–zeolite_/h needed for industrial applicability.^45,102,103^ While the current values derived from Cu-ERI zeolites do not yet reach the necessary industrial productivity, these results represent significant progress, considering the high selectivity toward methanol in the chemical looping process. Moreover, reducing the timeframes for each step in the loop by tuning the material compositions (e.g., lowering the Si/Al ratio and adjusting the copper species) and optimizing process design is expected to further enhance the potential productivity.^45^

## Conclusions

In summary, this work presents a systematic study of the complex nature of copper species hosted in Cu-ERI zeolites for the direct conversion of methane to methanol. We demonstrate that the compositional characteristics (Cu/Al and Si/Al ratios) of Cu-ERI zeolites govern the redox properties of copper species and the methanol yield. Using operando XAS, we were able to directly probe the evolution of copper species during all steps of the chemical looping process. The results from operando XAS, in combination with LCF analysis, reveal that the Cu-ERI zeolite with a higher Cu/Al ratio possesses higher reducibility. However, adding more copper does not always result in a higher methanol yield. While the Si/Al ratio appears not to affect the reducibility of copper species or the normalized methanol yield at a given Cu/Al ratio, increasing the Al content enhances the total methanol yield. The potential productivity of Cu-ERI-4.6(0.30) is estimated to ∼785 μmol/g_–zeolite_/h, approximately a quarter of the industrially desirable target of 3000 μmol/g_–zeolite_/h. These results contribute to a deeper understanding of how the composition affects the performance of copper-exchanged zeolites in the direct oxidation of methane to methanol. Furthermore, zeolite selection plays a pivotal role in determining the nuclearity, accessibility, and reactivity of copper species. Based on the findings, zeolite structures featuring cages with limited openings, such as AEI, LTA, and DDR, may serve as promising candidates for further investigation. The insights gained from this work could inform the design of more efficient materials for methane conversion, potentially leading to improved processes to produce methanol from natural gas.

## Supplementary Material



## Notes

Additional details of experimental procedures, sample preparation, and characterization of Cu-ERI materials; XRD patterns, SEM images, and FTIR spectra of NO adsorption; Cu K-edge XANES, *k* ^2^-weighted FT EXAFS data, and the fitted spectra of Cu-ERI materials collected after each reaction step; linear combination fitting analysis of TPR-CH_4_ (PDF)
