# Supplementary material for: Elucidation of the Structure–Activity Relationship for Cu-Erionite in the Direct Conversion of Methane to Methanol: An Operando XAS Study
Source: J Am Chem Soc. 2025 Jun 20;147(32):28723–34. doi: 10.1021/jacs.5c03554 (PMC12356592; doi:10.1021/jacs.5c03554)
Supplement: Supplementary file 1 [file ja5c03554_si_001.pdf]

Supporting Information

Elucidation of the Structure–Activity Relationship for Cu-Erionite in the Direct Conversion of Methane to Methanol: an Operando XAS Study

Jie Zhu,[†‡§] Vitaly L. Sushkevich,[*‡] Amy J. Knorpp,[†] Mark A. Newton,[†#] Toru Wakihara,[§Φ] Tatsuya Okubo,[§] Zhendong Liu[*⊥§Φ] and Jeroen A. van Bokhoven[*†‡]

[†] Institute for Chemical and Bioengineering, ETH Zurich, Vladimir-Prelog-Weg 1, 8093 Zurich, Switzerland

[‡] Center for Energy and Environmental Sciences, Paul Scherrer Institute, 5232 Villigen PSI, Switzerland

[#]Department of Structure and Dynamics in Catalysis, J. Heyrovsky Institute of Physical Chemistry, Dolejškova 2155/3, 182 23 Prague 8, Czech Republic

[§] Department of Chemical System Engineering, The University of Tokyo, 7-3-1 Hongo, Bunkyo-ku, Tokyo 113-8656, Japan

[Φ]Institute of Engineering Innovation, The University of Tokyo, 2-11-16 Yayoi, Bunkyo-ku, Tokyo 113-8656, Japan

[⊥]State Key Laboratory of Chemical Engineering, Department of Chemical Engineering, Tsinghua University, Haidian District, Beijing 100084, China

Contents

1. Experimental Section

1.1 Synthesis of Cu-ERI Zeolites with Different Cu/Al and Si/Al Ratios
1.2 Characterization
1.3 Methane Oxidation Reaction over Cu-ERI Materials
1.4 Operando X-ray Absorption Spectroscopy (XAS)
1.5 Data Analysis and Fitting
1.6 Calculations

2. Supplementary Tables

Table S1. EXAFS Fitting Results of Cu-ERI Zeolites with Different Si/Al Ratios after Activation in Oxygen at 450 °C for 1 h

Table S2. EXAFS Fitting Results of Cu-ERI Zeolites with Different Si/Al Ratios after Reaction with Methane at 300 °C for 30 min

Table S3. Summary of the Influence of Cu/Al and Si/Al Ratios on Methanol Productivity Achieved Over Various Zeolite Topologies

3. Supplementary Figures

Figure S1. Powder X-ray diffraction patterns of Cu-ERI-6.4(*y*) zeolites with different Cu/Al ratios

Figure S2. SEM images of Cu-ERI-*x*(0.30) zeolites with different Si/Al ratios

Figure S3. FTIR spectra of nitrogen monoxide adsorbed over Cu-ERI-6.4(0.30) at 77 K within the pressure range of 0.01-0.3 torr

Figure S4. Quantitative analysis of FTIR spectra of Cu-ERI zeolites with different Cu/Al ratios

Figure S5. FTIR spectra of nitrogen monoxide over the Cu-ERI-6.4(0.30) zeolite after activation in oxygen at 400 °C and after reaction with methane at 300 °C

Figure S6. Scheme for in situ XAS measurement during the conversion of methane to methanol over Cu-ERI zeolites

Figure S7. Cu K-edge XANES of Cu-ERI-6.4(*y*) zeolites with Cu/Al ratios of 0.21,

0.30 and 0.41 after each step in the conversion of methane to methanol

Figure S8. k^2-weighted $\chi(k)$ data acquired over activated Cu-ERI-6.4(y) zeolites with different Cu/Al ratios at 450 °C

Figure S9. Fitting results of k^2-weighted FT EXAFS spectrum of activated Cu-ERI-6.4(0.11)

Figure S10. Fitting results of k^2-weighted FT EXAFS spectrum of activated Cu-ERI-6.4(0.21)

Figure S11. Fitting results of k^2-weighted FT EXAFS spectrum of activated Cu-ERI-6.4(0.30)

Figure S12. Fitting results of k^2-weighted FT EXAFS spectrum of activated Cu-ERI-6.4(0.41)

Figure S13. k^2-weighted $\chi(k)$ data acquired over Cu-ERI-6.4(y) zeolites different Cu/Al ratios after reaction with 1 bar of methane at 300 °C

Figure S14. Fitting results of k^2-weighted FT EXAFS spectrum of Cu-ERI-6.4(0.21) after reaction with 1 bar of methane

Figure S15. Fitting results of k^2-weighted FT EXAFS spectrum of Cu-ERI-6.4(0.30) after reaction with 1 bar of methane

Figure S16. Fitting results of k^2-weighted FT EXAFS spectrum of Cu-ERI-6.4(0.41) after reaction with 1 bar of methane

Figure S17. k^2-weighted $\chi(k)$ data acquired over activated Cu-ERI-x(0.30) zeolites with different Si/Al ratios at 450 °C

Figure S18. Fitting results of k^2-weighted FT EXAFS spectrum of activated Cu-ERI-4.6(0.30)

Figure S19. Fitting results of k^2-weighted FT EXAFS spectrum of activated Cu-ERI-9.1(0.30)

Figure S20. k^2-weighted $\chi(k)$ data acquired over Cu-ERI-x(0.30) zeolites different Si/Al ratios after reaction with 1 bar of methane at 300 °C

Figure S21. Fitting results of k^2-weighted FT EXAFS spectrum of Cu-ERI-4.6(0.30) after reaction with 1 bar of methane

Figure S22. Fitting results of k^2-weighted FT EXAFS spectrum of Cu-ERI-9.1(0.30) after reaction with 1 bar of methane

Figure S23. Linear combination fitting (LCF) of XANES spectra obtained over Cu-ERI-6.4(0.11) upon the reaction with 1 bar of methane at 300 °C

Figure S24. Logarithmic plots of Cu^I species formed over Cu-ERI-6.4(*y*) zeolites with different Cu/Al ratios during the reaction with 1 bar of methane

Figure S25. Cu K-edge XANES of Cu-ERI-6.4(*y*) zeolites with different Cu/Al ratios during reaction under different methane pressure

Figure S26. Dependence of Cu^I fraction on methane pressure over Cu-ERI-6.4(*y*) zeolites with different Cu/Al ratios

Figure S27. LCF analysis of XANES spectra acquired during TPR-CH_4 of Cu-ERI-*x*(0.30) zeolites with different Si/Al ratios

Figure S28. Cu K-edge XANES of Cu-ERI with different Cu/Al ratios after reaction with 15 bar of methane and that after extraction with water

Figure S29. Cu K-edge XANES of Cu-ERI-6.4(0.30) after reaction with 1bar methane at 300 °C for 30 min for three cycles

1. Experimental Section

1.1 Synthesis of Cu-ERI Zeolites with Different Cu/Al and Si/Al Ratios

The ERI zeolite with Si/Al=6.4 was synthesized according to the procedure reported in a previous work.[1] The initial reactant mixture had a composition of 1.63 RBr_2: 7.8 KOH: 0.8 Al_2O_3: 16 SiO_2: 258 H_2O, where RBr_2 denotes hexamethonium bromide. In particular, aluminum *sec*-butoxide ($Al[OCH(CH_3)C_2H_5]_3$, 97%, Aldrich) was dissolved in KOH (Wako Pure Chemical Industries, Ltd., 30 wt%) solution, followed by the dropwise addition of a solution containing hexamethonium bromide (Tokyo Chemical Industry, Ltd., >98 wt%) dissolved in de-ionized water. Then colloidal silica (LUDOX® AS-40, 40 wt.% suspension) was added slowly to form an aluminosilicate reactant. The reactant mixture was homogenized for 2 h and aged for 20 h at 95 °C in an oven. To this aluminosilicate mixture, 10 wt% seed crystals synthesized through the charge density mismatch (CDM) approach were added,[1] and the resulting reactant was then stirred for 10 min prior to the hydrothermal treatment. Finally, 1.7 g of the reactant mixture was fed into a tubular reactor (4.4 mm inner diameter, 6.6 mm outer diameter, 13.5 cm length) and heated at 210 °C for 2 h. The ERI product with Si/Al ratio=4.6 was synthesized from a reactant mixture having a composition of 1.63 RBr_2: 7.8 KOH: 1.6 Al_2O_3: 16 SiO_2: 258 H_2O. The synthesis procedure was the same as that for the synthesis of ERI with Si/Al=6.4.

For the synthesis of ERI with Si/Al=9.1, the initial reactant mixture had a composition of 1.63 RBr_2: 7.8 KOH: 0.27 Al_2O_3: 16 SiO_2: 258 H_2O, where CBV 760 (dealuminated Y zeolite with Si/Al=31, Zeolyst International) was used as starting material. Firstly, CBV 760 zeolite powder was dissolved in KOH solution, followed by the dropwise addition of a solution containing hexamethonium bromide dissolved in de-ionized water. De-ionized water was then added slowly to form an aluminosilicate reactant. The reactant mixture was homogenized for 2 h and aged for 20 h at 95 °C in an oven. To this aluminosilicate mixture, 10 wt% seed crystals synthesized *via* the CDM method were added, and the resulting reactant was then stirred for 10 min prior to the hydrothermal treatment. Finally, 1.7 g of the reactant mixture was fed into a tubular reactor (4.4 mm inner diameter, 6.6 mm outer diameter, 13.5 cm length) and heated at 210 °C for 4 h.

The synthesized ERI zeolites were calcined at 550 °C for 8 h to remove the organic structure-directing molecules occluded in the zeolites. The calcined ERI zeolites were then ion-exchanged twice with 1 M NH_4NO_3 solution at 90 °C for 5 h. Cu-ERI materials were prepared by liquid ion exchange of NH_4-ERI with $(CH_3COO)_2Cu$ solution at 90 °C for 30 min. After centrifugation and drying in an oven, the obtained Cu-ERI zeolites were calcined at 550 °C for 3 h. Cu-ERI materials with different copper loadings were readily

controlled by varying the concentration of $(CH_3COO)_2Cu$ aqueous solutions.

1.2 Characterization

Powder X-ray diffraction (XRD) patterns of the catalysts were collected with a Panalytical X'Pert Pro MPD instrument (λ = 0.15406 nm, 40 kV, 40 mA) at a scan rate of 4 °/min. Elemental analysis of the products was carried out using a SpectraAA 220FS atomic absorption spectrometer. Fifteen milligrams of Cu-ERI were dissolved with 2 ml hydrofluoric acid and 3 ml nitric acid and then diluted to 50 ml with deionized water. FTIR spectroscopy was performed on a Thermo Nicolet iS50 spectrometer with a MCT detector. Optical adsorption data were collected from 400 to 4000 cm^{-1} at a resolution of 4 cm^{-1} and with 128 scans. The Cu-ERI sample (20 mg) was pressed into a self-supporting disc and activated in the IR cell with 300 torr of oxygen at 400 °C for 1 h. The system was then evacuated and cooled to 77 K with liquid nitrogen. Nitrogen monoxide (NO) was dosed into the cell with the subsequent acquisition of the IR spectrum. The dosing was stopped until the saturation of the sample with NO. Spectra of surface species were obtained by subtracting the reacted sample from the activated Cu-ERI samples with the OMNIC 9.1 software package.

1.3 Methane Oxidation Reaction over Cu-ERI Materials

Methane oxidation reaction measurements were carried out in a high-pressure reactor. Approximately 300 mg of Cu-ERI material was loaded into a stainless-steel autoclave. The sample was activated in a pure flow of O_2 at 450 °C for 1 h. The reactor system was then cooled to 300 °C and purged with a pure helium flow for 20 min. Then, pure methane was fed into the reactor at the same temperature. After the reaction with methane at 300 °C for 30 min, the reactor was then cooled down to room temperature with a flow of pure helium. The obtained methanol was extracted offline by adding 2 mL de-ionized water to the reacted Cu-ERI sample, and the resulting suspension was stirred for 1 h at room temperature. The aqueous suspension was then filtered and analyzed with a gas chromatograph (Agilent 6890). Butanol was added as the external standard solution. Multiple extractions were performed on Cu-ERI samples for the calculation of methanol yield, where the solid samples were collected after the previous extraction and the process was repeated until no more methanol was detected by the gas chromatograph.

1.4 Operando X-ray Absorption Spectroscopy (XAS)

XAS measurements were carried out at the XAFS beamline at the Elettra Sincrotrone Trieste, Italy. Cu-ERI zeolites were ground into fine powder and packed between two

quartz wools in a 1.5 mm quartz capillary reactor with 0.1 mm wall thickness. The spectra were collected in transmission mode with ionization chambers for the detection of incident and transmitted photons. Around 13-15 mg Cu-ERI zeolite was loaded in the reactor and activated in a pure flow of O_2 at 450 °C with a heating ramp of 10 °C/min. After the activation, the system was cooled down to 300 °C and purged with a pure He flow for 20 min. Then methane flow was introduced to the capillary reactor for 30 min. The reactor was then cooled down to 200 °C in a pure He flow and followed by the extraction with steam (10% H_2O/He) for 30 min. The XANES spectra were recorded through the complete chemical looping process. EXAFS spectra were collected during different stages of the process. For temperature-programmed reaction with methane (TPR-CH_4) measurement, Cu-ERI zeolite was first activated in O_2 with the same conditions described above. Next, the sample was purged with He flow and cooled down to 50 °C. A flow of CH_4 was subsequently introduced to the capillary reactor, followed by heating with a rate of 10 °C/min to 600 °C. XANES spectra were collected every 50 °C, which enabled us to perform linear combination fitting (LCF) to compare the reducibility of copper species in Cu-ERI samples. The spectra obtained after the activation in O_2 and the one recorded after reacting with CH_4 at 600 °C were employed as the standards for Cu^{II} and Cu^{I}, respectively.

High quality EXAFS spectra of the activated Cu-ERI samples with different compositions (Cu/Al and Si/Al ratios) were collected in transmission mode using nitrogen-filled ionization chamber detectors at the Swiss Light Source (SLS). The samples were loaded into thin-walled capillaries and activated in flowing oxygen at 450 °C for 1h, followed by evacuation to remove any residual species. Each Cu-ERI sample was placed between the first and second ionization chambers, cooled down to -140 °C using a cold nitrogen vapor jet and measured to obtain 1200 single spectra for each sample.

1.5 XAS Data Analysis and Fitting

XAS data was analyzed using Athena software from Demeter package. The normalization of the spectra and background subtraction were processed in Athena program with copper foil as reference. The Fourier transform (FT) fitting of the EXAFS spectra was fitted using Artemis software. Phase and amplitude were calculated with FEFF6 code. k^2-weighted $\chi(k)$ EXAFS spectra were transformed in the k range of 2.4 to 10, with the fitting in R-space of 1.0-3.2 Å region. The amplitude factor was estimated as 0.91 by the fitting the first-shell of the copper foil standard, which was used throughout the analysis.

1.6 Calculations

The total methanol yield and copper-normalized methanol yield are calculated using the following equations, where n denotes the molar quantity.

$$\text{Total methanol yield (μmol/g-zeolite)}=\frac{n(\text{MeOH})}{\text{Mass of zeolite}} \quad \text{Eq. (1)}$$

$$\text{Copper-normalized methanol yield (mol/mol-Cu)}=\frac{n(\text{MeOH})}{n(\text{Cu})} \quad \text{Eq. (2)}$$

The potential productivities that could be derived from Cu-ERI zeolites with different compositions are calculated based on the time required to reduce 50% of Cu^{II} in each sample, and the estimated desorption and reoxidation times—both of which are based on observations in previous work—set at 400 s and equal to the reduction time, respectively.[2] The productivities are determined using the following definition.

$$\text{Potential productivity (μmol/g-zeolite/h)} = \frac{n(\text{MeOH})}{(t1_{reduction} + t2_{desorption} + t3_{reactivation}) \times \text{Mass of zeolite}} \quad \text{Eq. (3)}$$

$t1_{reduction}$, $t2_{desorption}$ and $t3_{reactivation}$ represent the time required to Cu^{II} reduction, methanol desorption, and Cu^{I} reoxidation, respectively.

2. Supplementary Tables

Table S1. EXAFS Fitting Results of Cu-ERI-*x*(0.30) Zeolites with Different Si/Al Ratios after Activation in Oxygen at 450 °C for 1 h

Sample	Path	CN	Distance
Cu-ERI-4.6(0.29)	Cu-O_{ss}	3.9±0.2	1.91±0.020
Cu-ERI-6.4(0.30)	Cu-O_{ss}	3.7±0.2	1.94±0.021
Cu-ERI-9.1(0.30)	Cu-O_{ss}	3.7±0.2	1.90±0.019

Table S2. EXAFS Fitting Results of Cu-ERI-*x*(0.30) Zeolites with Different Si/Al Ratios after Reaction with Methane at 300 °C for 30 min

Sample	Path	CN	Distance
Cu-ERI-4.6(0.29)	Cu-O_{ss}	1.6±0.2	1.92±0.040
Cu-ERI-6.4(0.30)	Cu-O_{ss}	1.6±0.2	1.93±0.043
Cu-ERI-9.1(0.30)	Cu-O_{ss}	1.5±0.1	1.93±0.032

Table S3. Summary of the Influence of Cu/Al and Si/Al Ratios on Methanol Productivity Achieved Over Various Zeolite Topologies

Zeolite	Si/Al ratio	Cu/Al ratio	Methanol yield (μmol/g-zeo)	Normalized yield (mol/mol-Cu)	References
Cu-MOR	7	0.18	170	0.47	3
	7	0.24	141	0.28	
	11	0.28	96	0.25	
	11	0.36	124	0.25	
Cu-MOR	10	0.05	35.7	0.23	4
	10	0.09	51.8	0.18	
	10	0.13	55.3	0.14	
	10	0.18	44.2	0.07	

Cu-MOR	11	0.07	~32	~0.31	5
	11	0.12	~50	~0.31	
	11	0.21	~90	~0.31	
	11	0.32	~135	~0.31	
	5.5	0.17	138	0.33	
	7.2	0.25	170	0.33	
	8.5	0.25	119	0.28	
	11	0.32	134	0.30	
	21	0.29	69	0.31	
Cu-MOR	6.5	0.38	-	0.14	6
	10	0.40	-	0.22	
	46	0.60	-	0.32	
Cu-MFI	14	0.20	40	0.19	7
	14	0.31	58	0.18	
	14	0.45	80	0.18	
	14	0.52	89	0.17	
	17	0.14	20	0.20	
	17	0.27	48	0.21	
	17	0.43	38	0.10	
	17	0.54	33	0.07	
Cu-CHA	12	0.14	-	0.10	8
	12	0.16	-	0.10	
	12	0.34	-	0.16	
	12	0.49	125	0.20	
	5	0.44	130	0.11	
	15	0.53	-	0.17	
	29	0.52	-	0.13	
Cu-MAZ	4.3	0.23	200	0.27	2, 9-10
	4.3	0.22	200	0.29	
	4.3	0.25	277	0.35	

3. Supplementary Figures

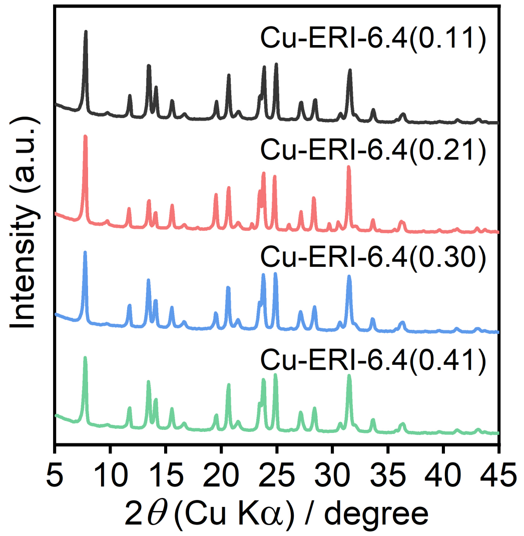

Figure S1. Powder X-ray diffraction patterns of Cu-ERI-6.4(y) zeolites with different Cu/Al ratios. y denotes the Cu/Al ratio of the samples.

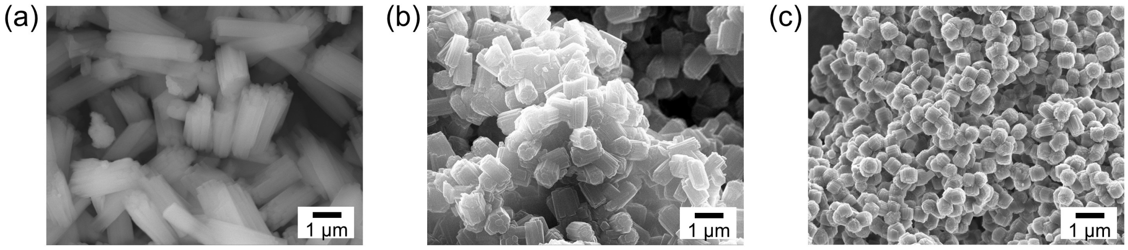

Figure S2. SEM images of (a) Cu-ERI-4.6(0.29); (b) Cu-ERI-6.4(0.30) and (c) Cu-ERI-9.1(0.30).

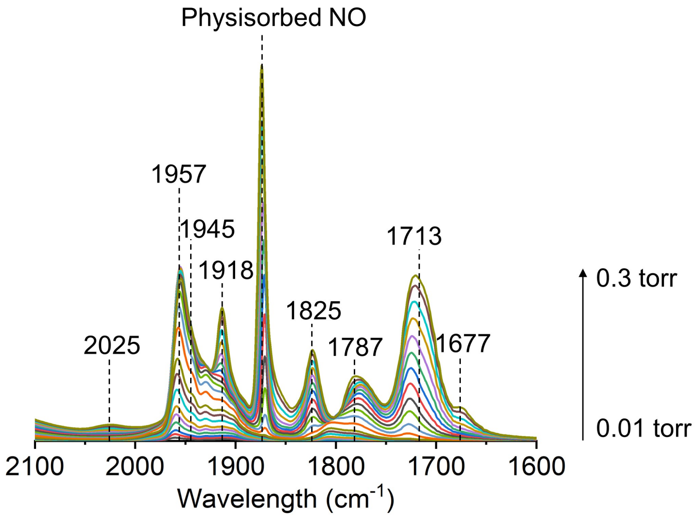

Figure S3. FTIR spectra of nitrogen monoxide adsorbed over Cu-ERI-6.4(0.30) at 77 K within the pressure range of 0.01-0.3 torr.

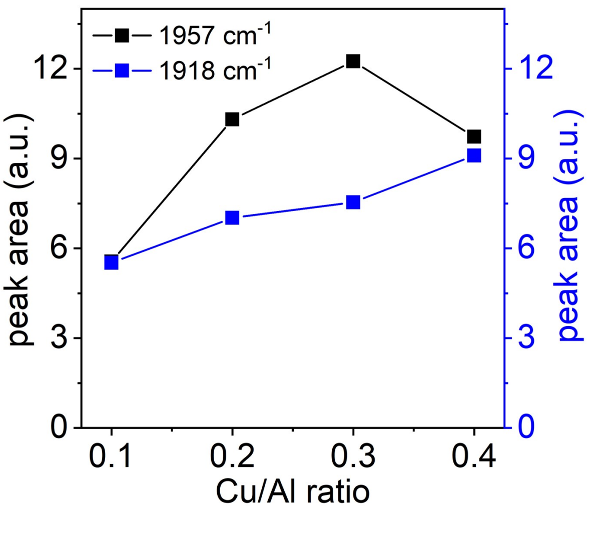

Figure S4. Quantitative analysis of the bands at 1957 cm^{-1} and 1918 cm^{-1} in FTIR spectra of Cu-ERI-6.4(*y*) zeolites with different Cu/Al ratios. *y* denotes the Cu/Al ratios.

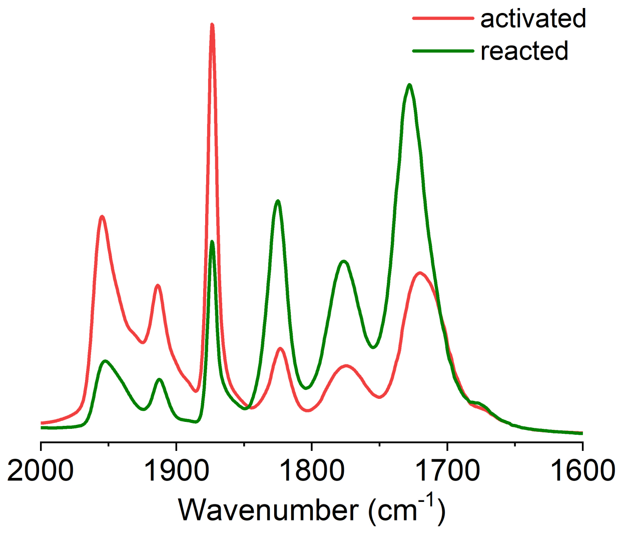

Figure S5. FTIR spectra of nitrogen monoxide over the Cu-ERI-6.4(0.30) zeolite after activation in oxygen at 400 °C (red) and after reaction with methane at 300 °C (green).

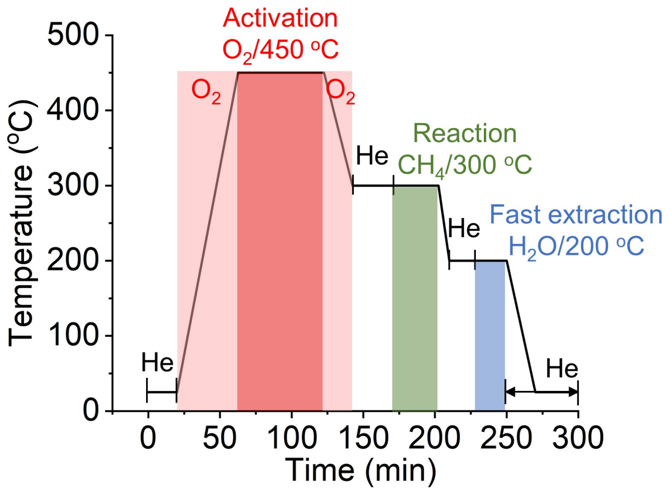

Figure S6. Scheme for in situ XAS measurement during the conversion of methane to methanol over Cu-ERI zeolites.

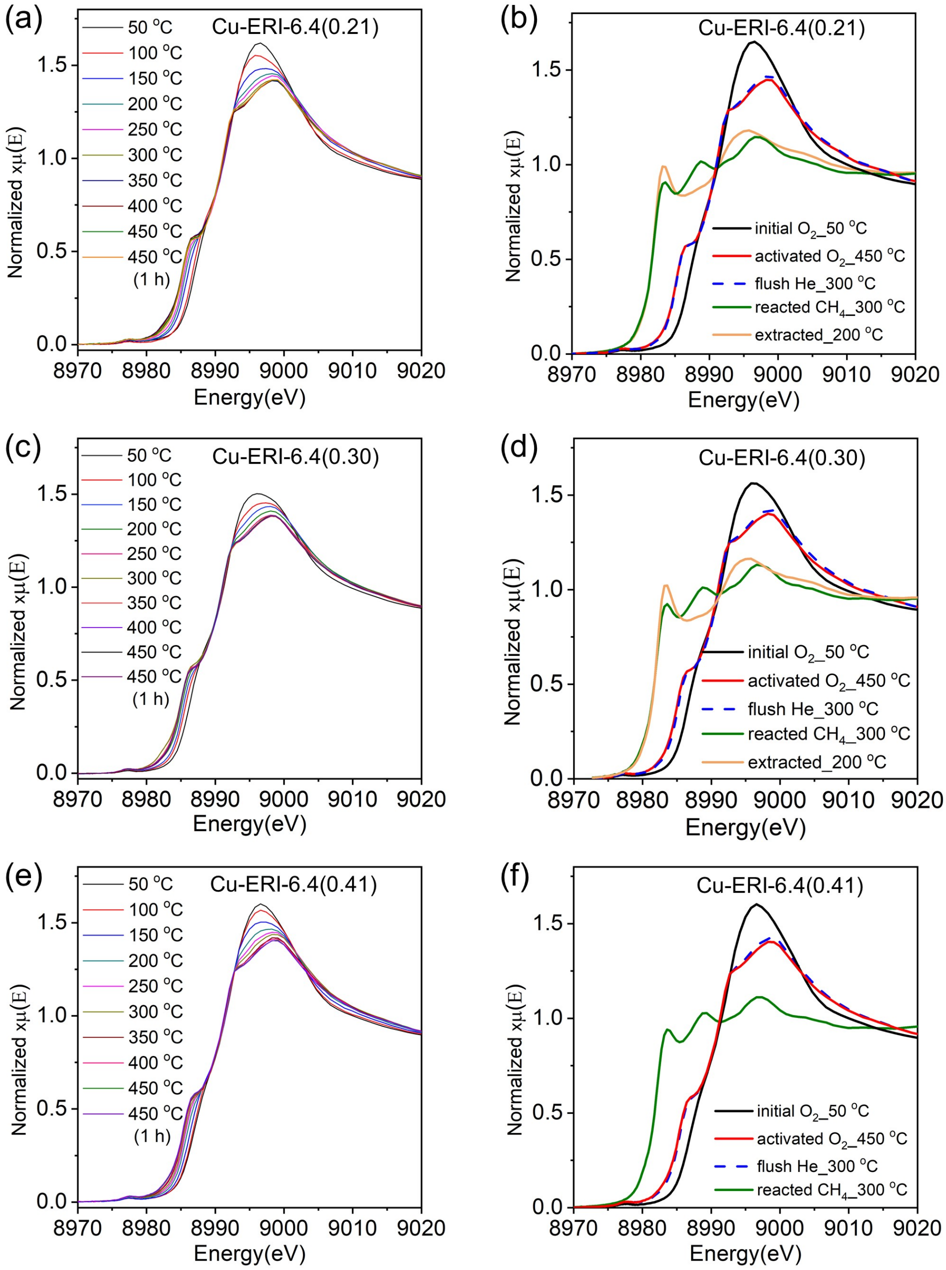

Figure S7. (a,c,e) Cu K-edge XANES of Cu-ERI-6.4(*y*) zeolites with Cu/Al ratios of 0.21, 0.30 and 0.41 during activation in oxygen for 1 h; (b,d,f) Cu K-edge XANES of Cu-ERI-6.4(*y*) zeolites with Cu/Al ratios of 0.21, 0.30 and 0.41 collected after each step in the conversion of methane to methanol: before activation at 50 °C, activation in oxygen at 450 °C, helium flush after cooling to 300 °C, reaction with methane at 300 °C, water-assisted extraction of methanol at 200 °C.

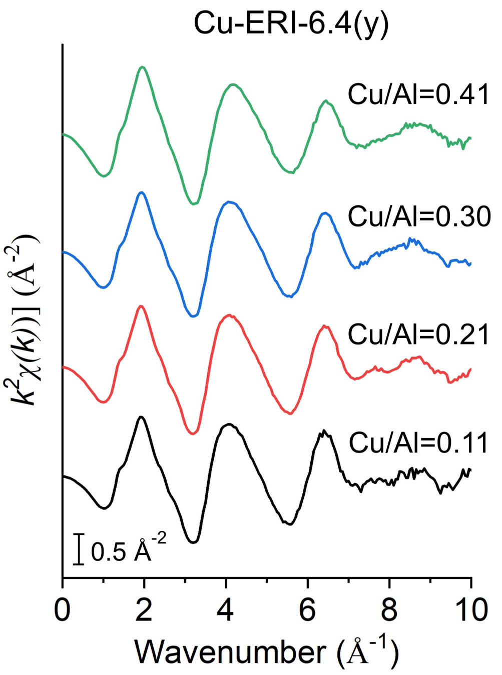

Figure S8. k^2-weighted $\chi(k)$ data acquired over activated Cu-ERI-6.4(y) zeolites with different Cu/Al ratios at 450 °C.

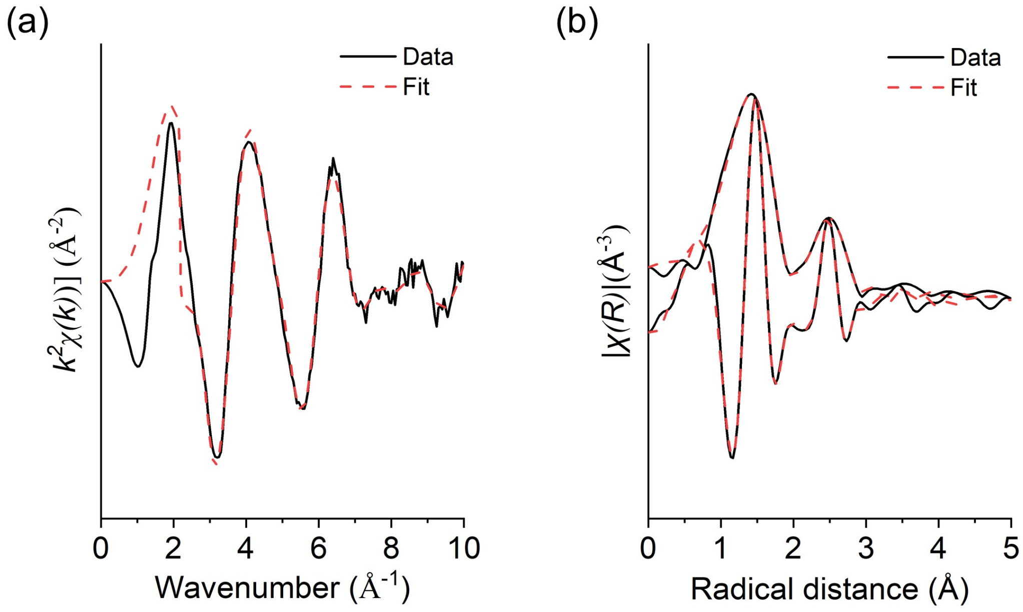

Figure S9. Fitting results of k^2-weighted FT EXAFS spectrum of activated Cu-ERI-6.4(0.11) in (a) k-space and (b) R-space magnitude and imaginary part. The fit was performed in the R range of 1.0–3.2 Å with employing the k-range of 2.4–10 Å^{-1}.

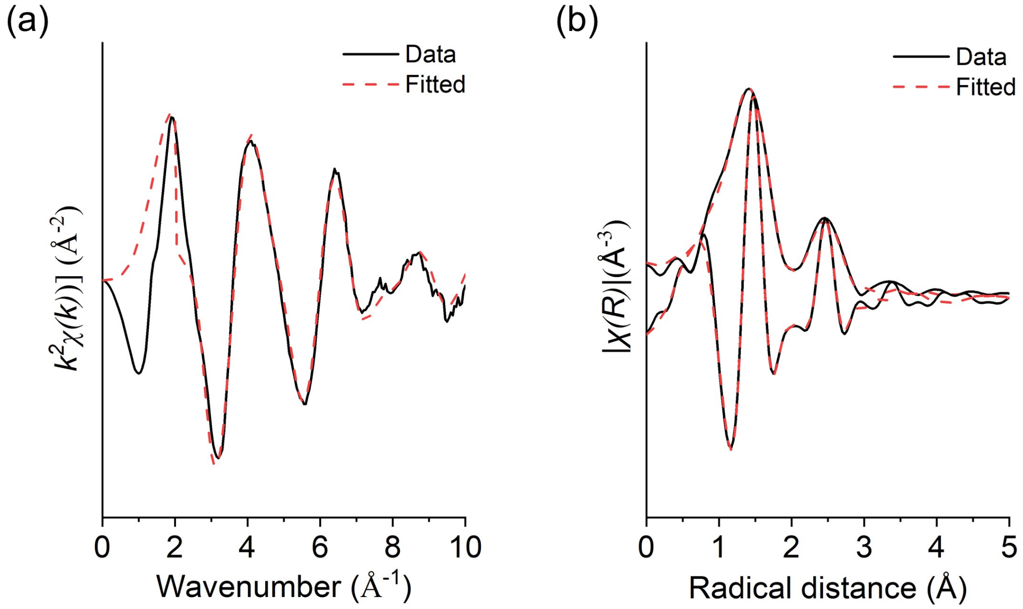

Figure S10. Fitting results of k^2-weighted FT EXAFS spectrum of activated Cu-ERI-6.4(0.21) in (a) *k*-space and (b) R-space magnitude and imaginary part. The fit was performed in the R range of 1.0–3.2 Å with employing the k-range of 2.4–10 Å^{-1}.

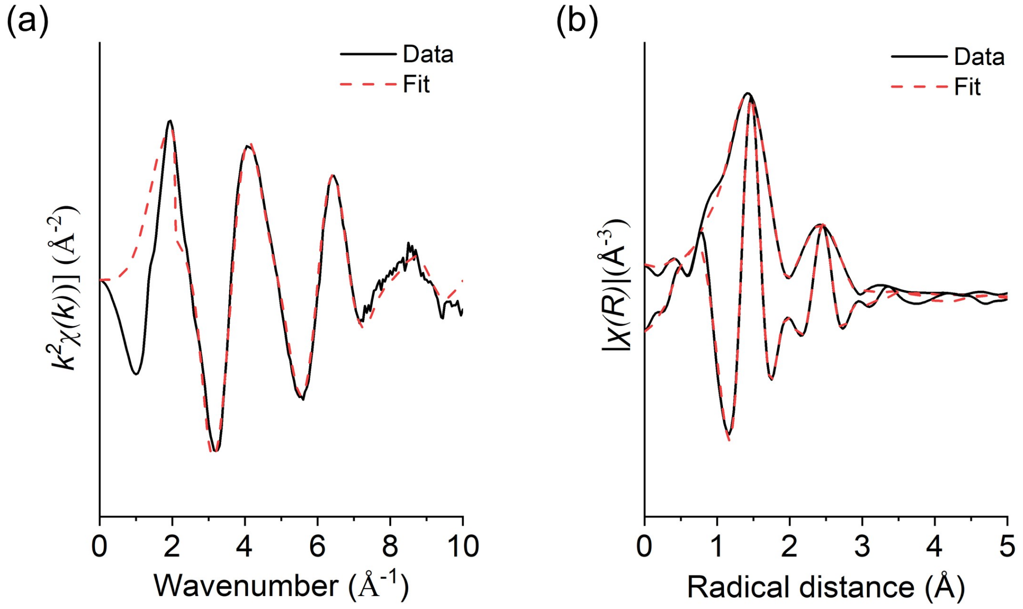

Figure S11. Fitting results of k^2-weighted FT EXAFS spectrum of activated Cu-ERI-6.4(0.30) in (a) *k*-space and (b) R-space magnitude and imaginary part. The fit was performed in the R range of 1.0–3.2 Å with employing the k-range of 2.4–10 Å^{-1}.

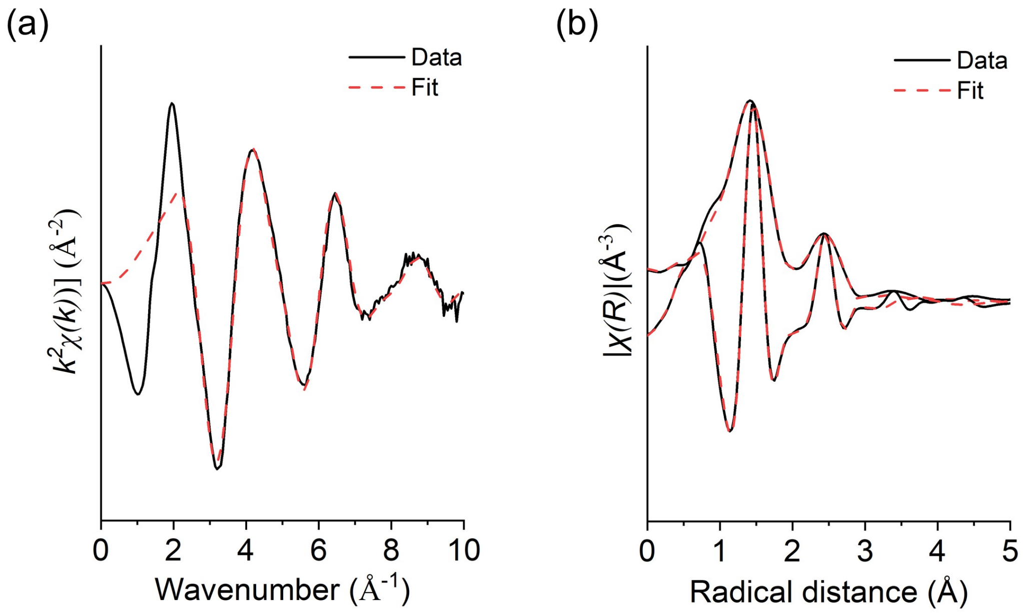

Figure S12. Fitting results of k^2-weighted FT EXAFS spectrum of activated Cu-ERI-6.4(0.41) in (a) *k*-space and (b) R-space magnitude and imaginary part. The fit was performed in the R range of 1.0–3.2 Å with employing the k-range of 2.4–10 $Å^{-1}$.

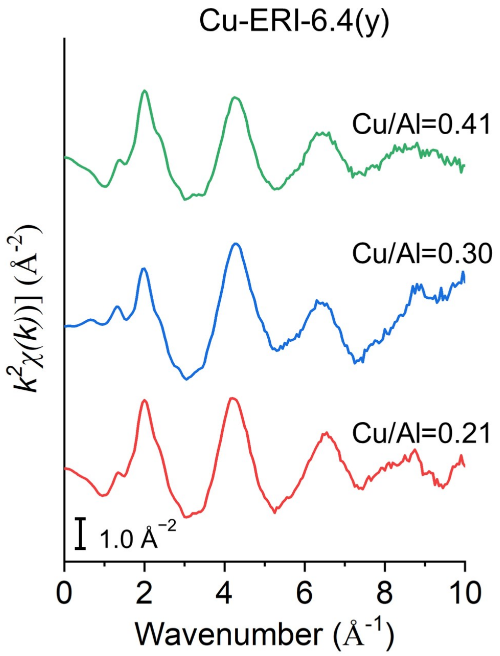

Figure S13. k^2-weighted $\chi(k)$ data acquired over Cu-ERI-6.4(*y*) zeolites different Cu/Al ratios after reaction with 1 bar of methane at 300 °C for 30 min.

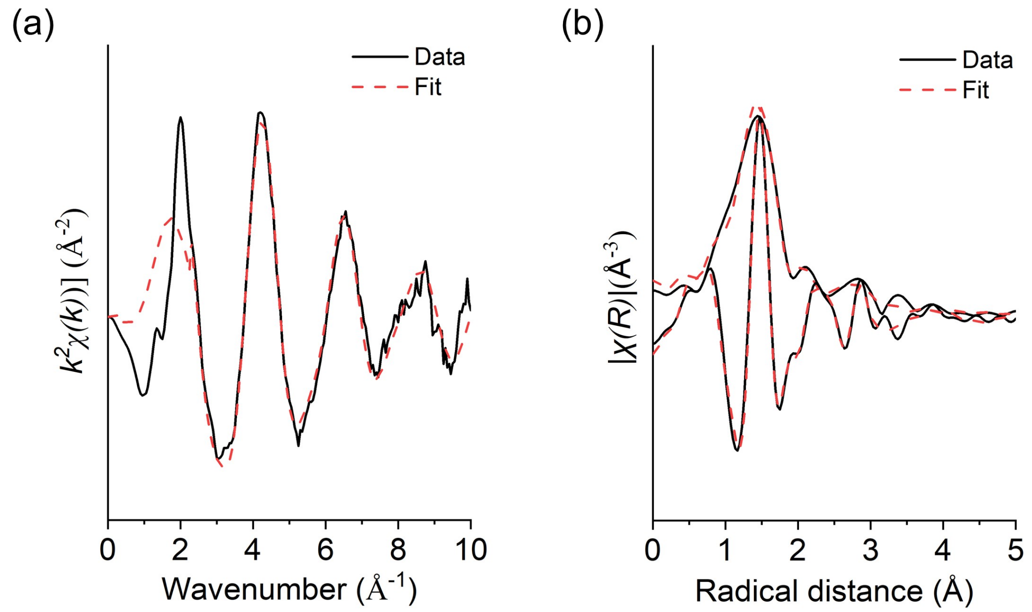

Figure S14. Fitting results of k^2-weighted FT EXAFS spectrum of Cu-ERI-6.4(0.21) after reaction with 1 bar of methane in (a) *k*-space and (b) R-space magnitude and imaginary part. The fit was performed in the R range of 1.0–3.2 Å with employing the k-range of 2.4–10 Å^{-1}.

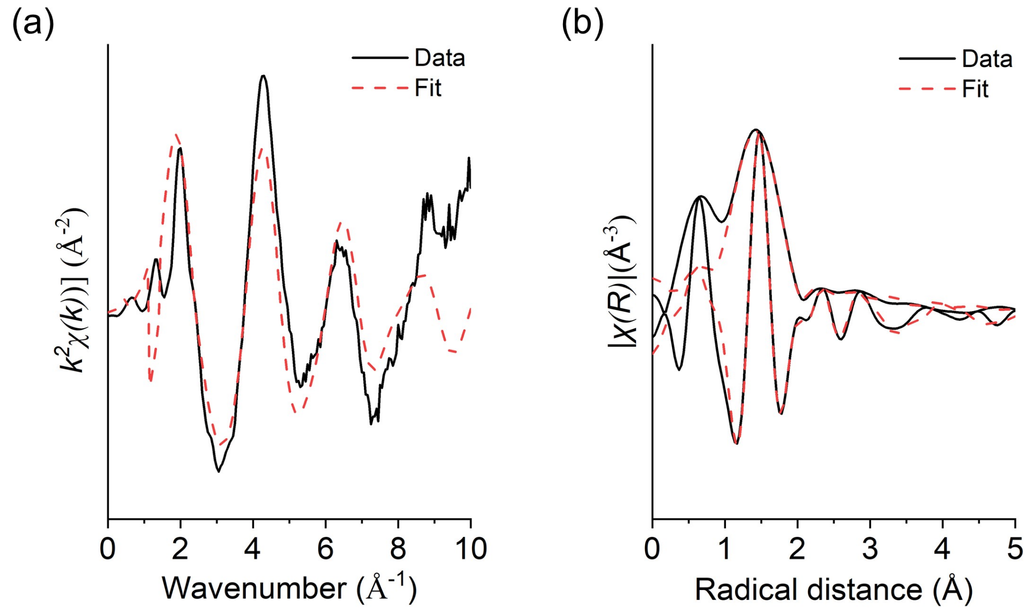

Figure S15. Fitting results of k^2-weighted FT EXAFS spectrum of Cu-ERI-6.4(0.30) after reaction with 1 bar of methane in (a) *k*-space and (b) R-space magnitude and imaginary part. The fit was performed in the R range of 1.0–3.2 Å with employing the k-range of 2.4–10 Å^{-1}.

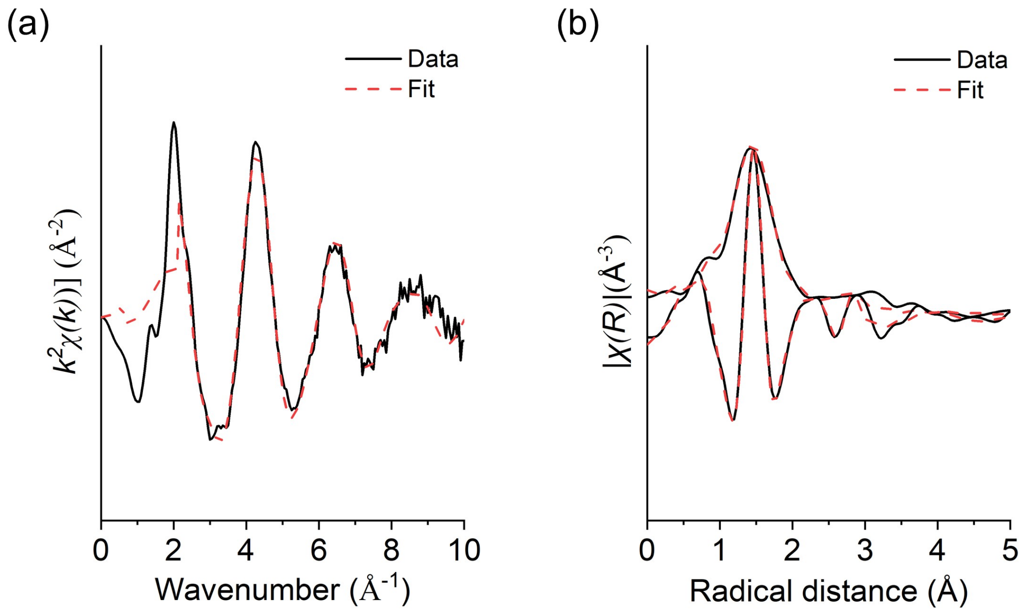

Figure S16. Fitting results of k^2-weighted FT EXAFS spectrum of Cu-ERI-6.4(0.41) after reaction with 1 bar of methane in (a) *k*-space and (b) R-space magnitude and imaginary part. The fit was performed in the R range of 1.0–3.2 Å with employing the k-range of 2.4–10 Å^{-1}.

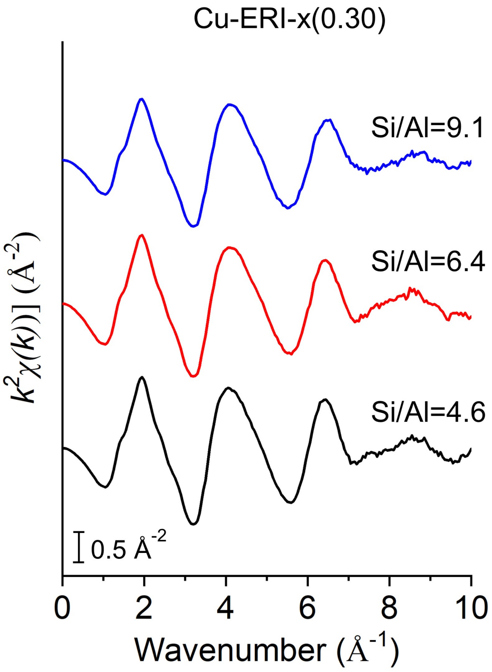

Figure S17. k^2-weighted χ(*k*) data acquired over activated Cu-ERI-*x*(0.30) zeolites with different Si/Al ratios at 450 °C.

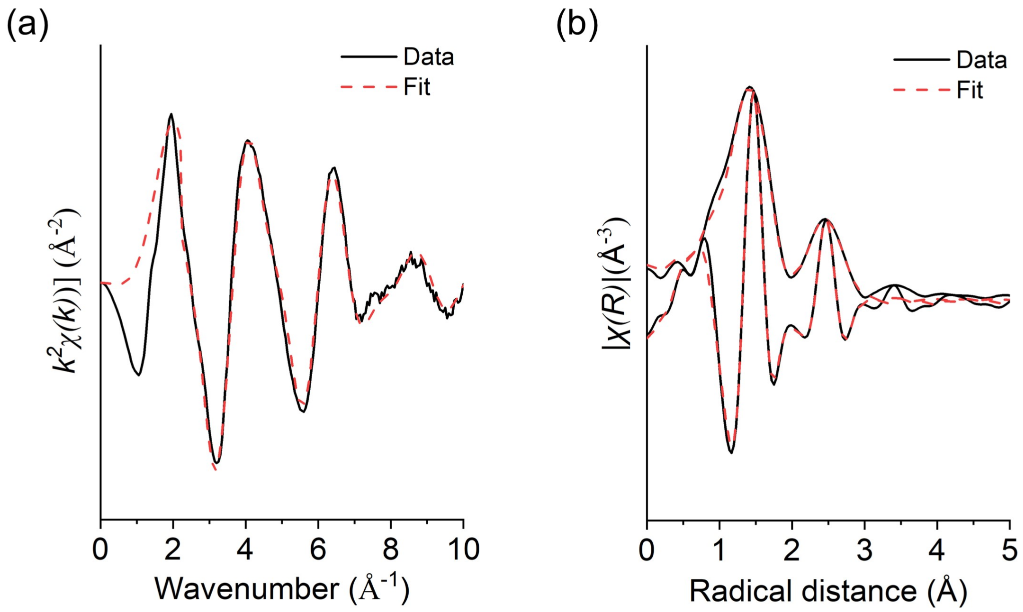

Figure S18. Fitting results of k^2-weighted FT EXAFS spectrum of activated Cu-ERI-4.6(0.30) in (a) *k*-space and (b) R-space magnitude and imaginary part. The fit was performed in the R range of 1.0–3.2 Å with employing the k-range of 2.4–10 $Å^{-1}$.

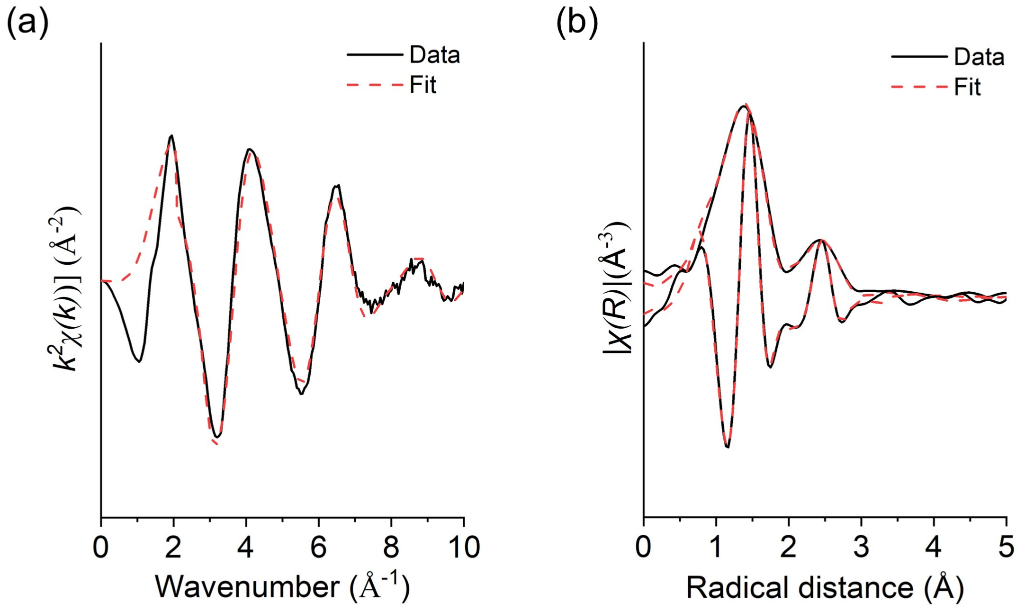

Figure S19. Fitting results of k^2-weighted FT EXAFS spectrum of activated Cu-ERI-9.1(0.30) in (a) *k*-space and (b) R-space magnitude and imaginary part. The fit was performed in the R range of 1.0–3.2 Å with employing the k-range of 2.4–10 $Å^{-1}$.

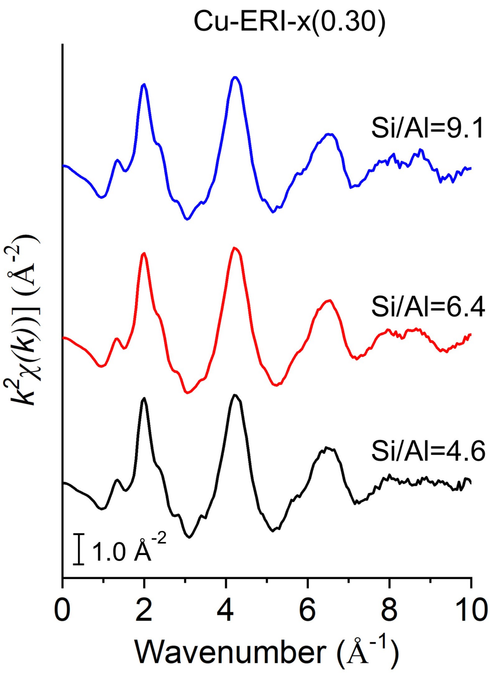

Figure S20. k^2-weighted χ(*k*) data acquired over Cu-ERI-*x*(0.30) zeolites different Si/Al ratios after reaction with 1 bar of methane at 300 °C for 30 min.

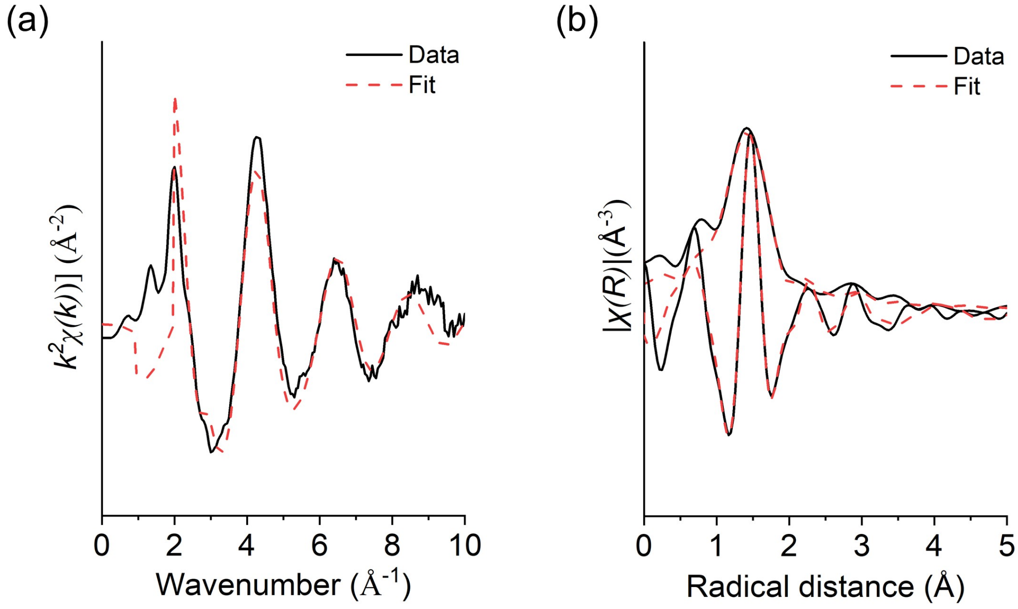

Figure S21. Fitting results of k^2-weighted FT EXAFS spectrum of Cu-ERI-4.6(0.30) after reaction with 1 bar of methane in (a) *k*-space and (b) R-space magnitude and imaginary part. The fit was performed in the R range of 1.0–3.2 Å with employing the *k*-range of 2.4–10 Å^{-1}.

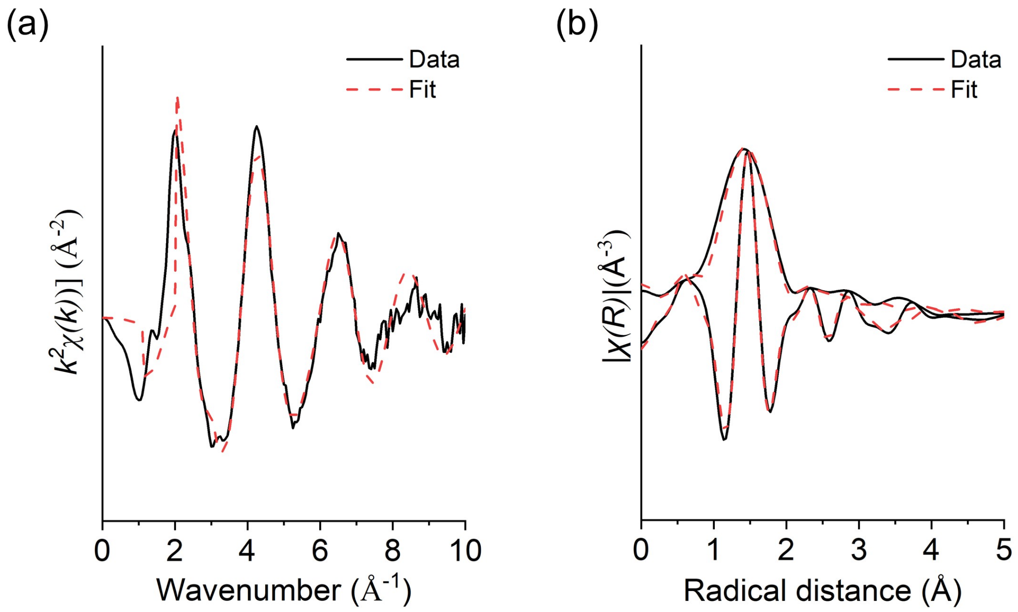

Figure S22. Fitting results of k^2-weighted FT EXAFS spectrum of Cu-ERI-9.1(0.30) after reaction with 1 bar of methane in (a) *k*-space and (b) R-space magnitude and imaginary part. The fit was performed in the R range of 1.0–3.2 Å with employing the *k*-range of 2.4–10 Å^{-1}.

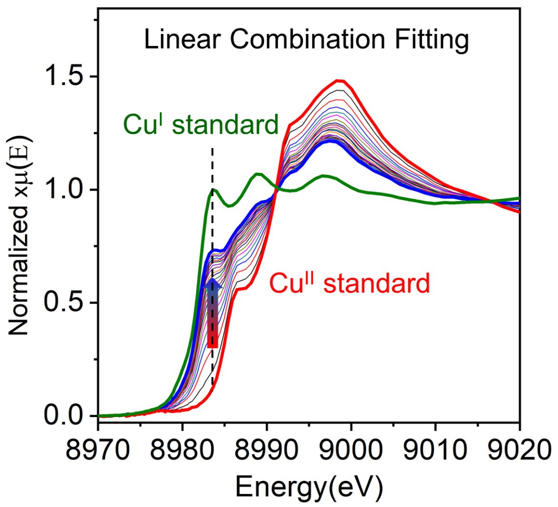

Figure S23. Linear combination fitting (LCF) of XANES spectra obtained over Cu-ERI-6.4(0.11) upon the reaction with 1 bar of methane at 300 °C. The spectrum collected after the activation in oxygen and the one recorded after reacting with methane at 600 °C were employed as Cu^{II} and Cu^{I} standard spectra, respectively.

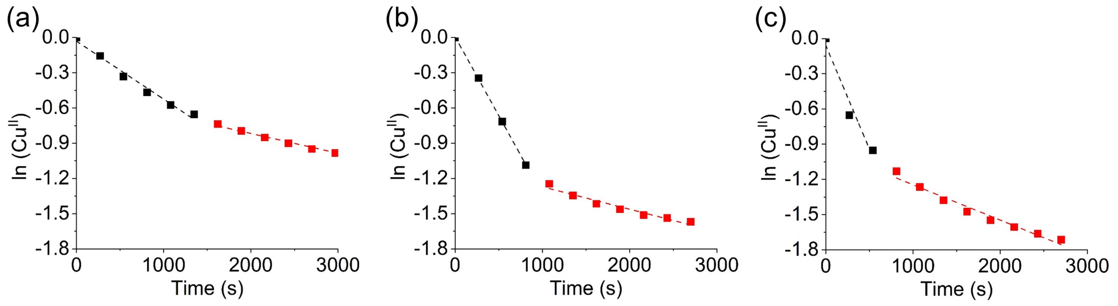

Figure S24. Logarithmic plots of Cu^{I} species formed over (a) Cu-ERI-6.4(0.11) (b) Cu-ERI-6.4(0.21) and (c) Cu-ERI-6.4(0.30) during the reaction with 1 bar of methane.

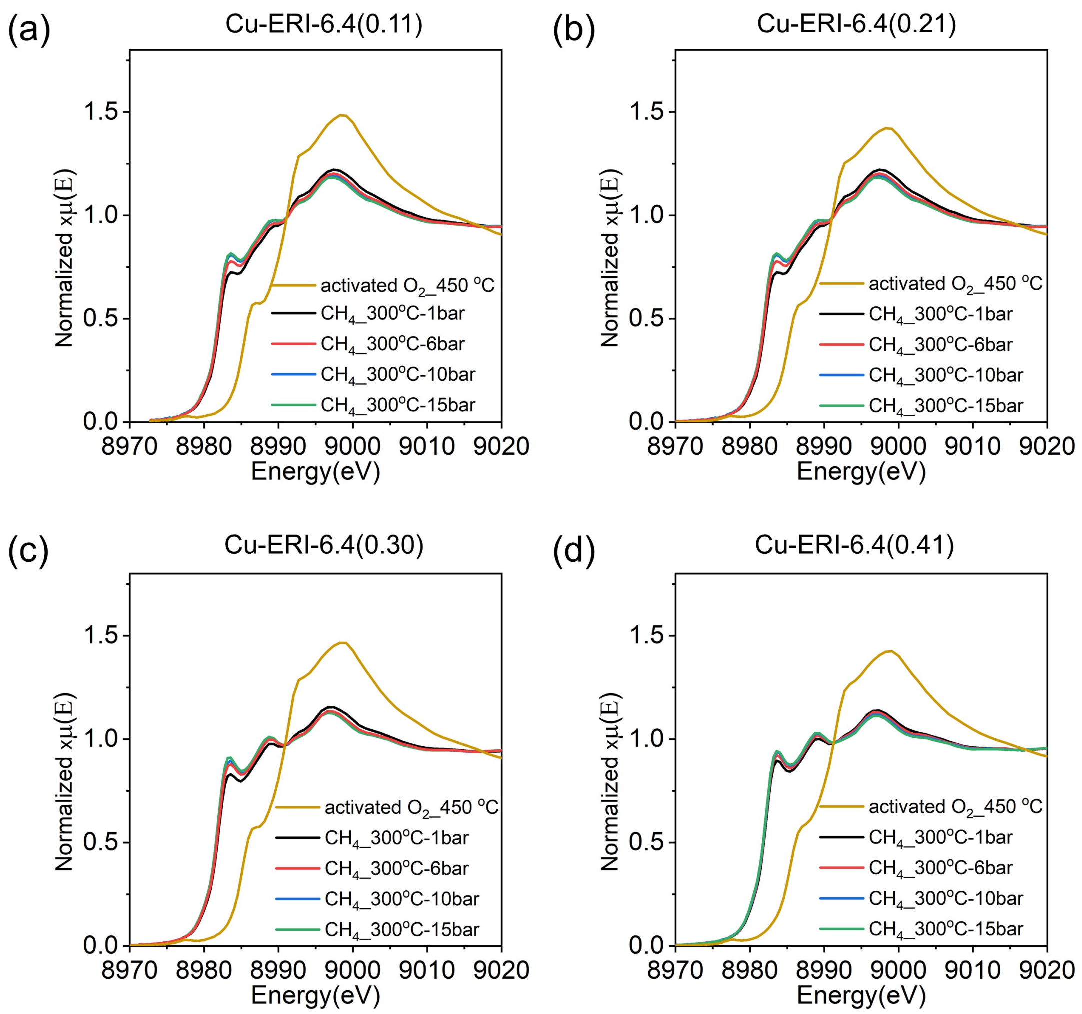

Figure S25. Cu K-edge XANES of (a) Cu-ERI-6.4(0.11) (b) Cu-ERI-6.4(0.21) Cu-ERI-6.4(0.30) and (d) Cu-ERI-6.4(0.41) during reaction under different methane pressures.

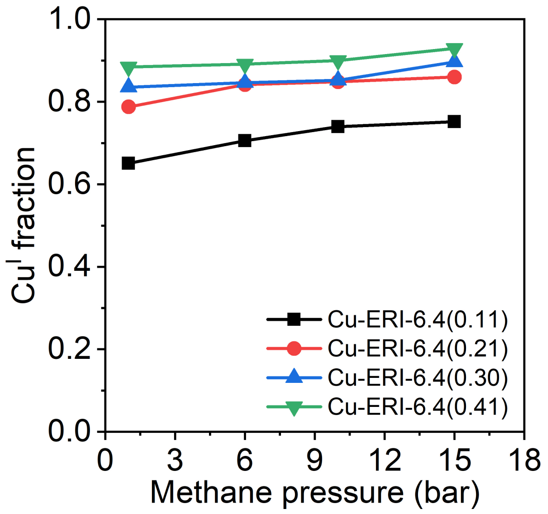

Figure S26. Dependence of Cu^I fraction on methane pressure over Cu-ERI-6.4(*y*) zeolites with different Cu/Al ratios.

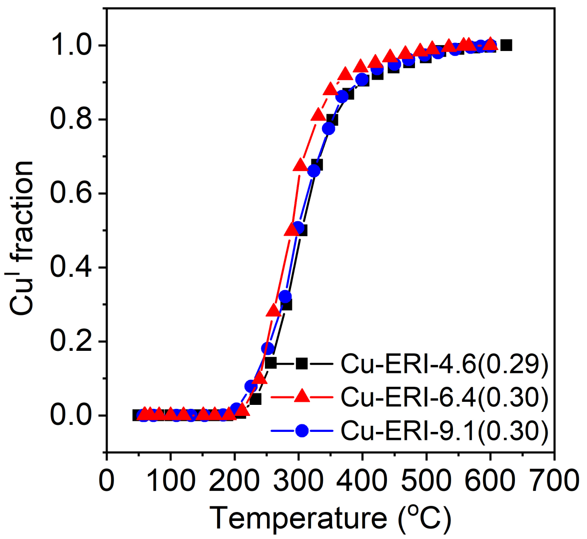

Figure S27. LCF analysis of XANES spectra acquired during TPR-CH_4 of Cu-ERI-*x*(0.30) zeolites with different Si/Al ratios.

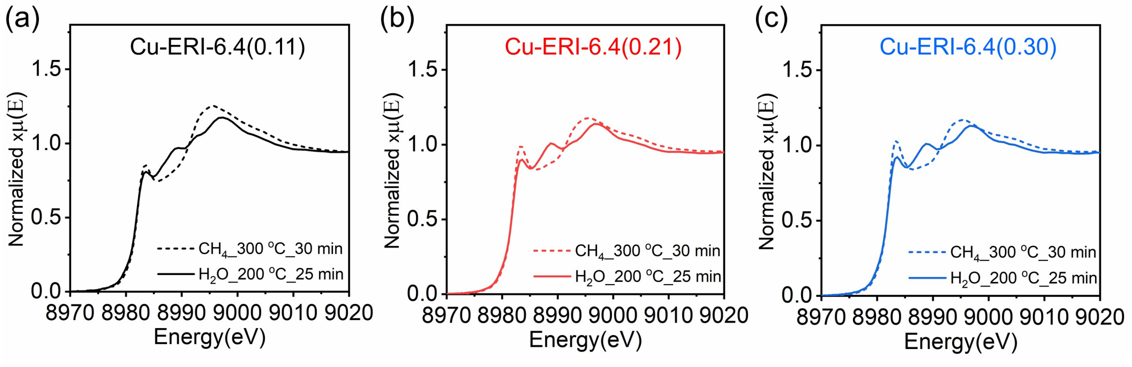

Figure S28. Cu K-edge XANES of Cu-ERI-6.4 with different Cu/Al ratios after reaction with 15 bar of methane and that after extraction with water.

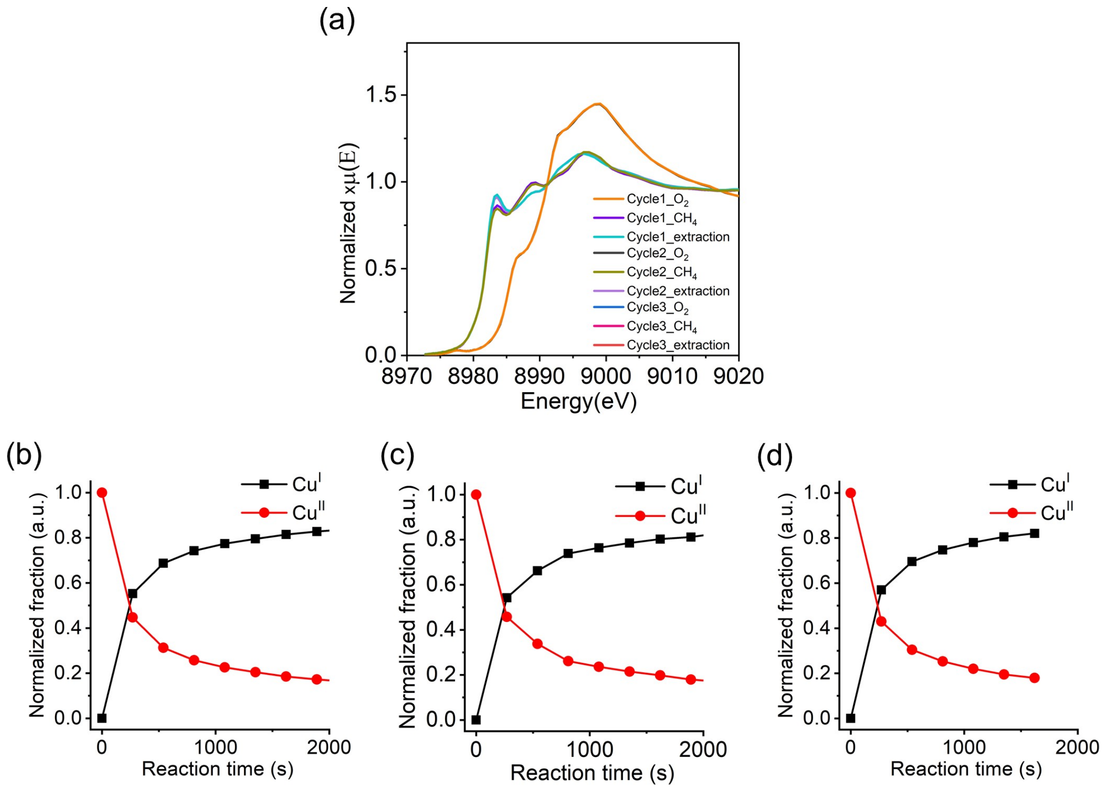

Figure S29. (a) Cu K-edge XANES of Cu-ERI-6.4(0.30) after reaction with 1bar methane at 300 °C for 30 min for three cycles; (b-d) LCF of XANES spectra obtained during the reaction of Cu-ERI-6.4(0.30) with 1 bar of methane for three cycles, (b) the first cycle; (c) the second cycle; (d) the third cycle.

References

1. Zhu, J.; Liu, Z.; Iyoki, K.; Anand, C.; Yoshida, K.; Sasaki, Y.; Sukenaga, S.; Ando, M.; Shibata, H.; Okubo, T.; Wakihara, T., Ultrafast Synthesis of High-Silica Erionite Zeolites with Improved Hydrothermal Stability. *Chem. Commun.* **2017,** *53* (50), 6796-6799.

2. Wieser, J.; Knorpp, A. J.; Stoian, D. C.; Rzepka, P.; Newton, M. A.; van Bokhoven, J. A., Assessing the Productivity of the Direct Conversion of Methane-to-Methanol over Copper-Exchanged Zeolite Omega (MAZ) via Oxygen Looping. *Angew. Chem. Int. Ed.* **2023,** *62* (40), e202305140.

3. Pappas, D. K.; Martini, A.; Dyballa, M.; Kvande, K.; Teketel, S.; Lomachenko, K. A.; Baran, R.; Glatzel, P.; Arstad, B.; Berlier, G.; Lamberti, C.; Bordiga, S.; Olsbye, U.; Svelle, S.; Beato, P.; Borfecchia, E., The Nuclearity of the Active Site for Methane to Methanol Conversion in Cu-Mordenite: A Quantitative Assessment. *J. Am. Chem. Soc.* **2018,** *140* (45), 15270-15278.

4. Le, H. V.; Parishan, S.; Sagaltchik, A.; Göbel, C.; Schlesiger, C.; Malzer, W.; Trunschke, A.; Schomäcker, R.; Thomas, A., Solid-State Ion-Exchanged Cu/Mordenite Catalysts for the Direct Conversion of Methane to Methanol. *ACS Catal.* **2017,** *7* (2), 1403-1412.

5. Grundner, S.; Markovits, M. A. C.; Li, G.; Tromp, M.; Pidko, E. A.; Hensen, E. J. M.; Jentys, A.; Sanchez-Sanchez, M.; Lercher, J. A., Single-site Trinuclear Copper Oxygen Clusters in Mordenite for Selective Conversion of Methane to Methanol. *Nat. Commun.* **2015,** *6* (1), 7546.

6. Sushkevich, V. L.; Palagin, D.; van Bokhoven, J. A., The Effect of the Active-Site Structure on the Activity of Copper Mordenite in the Aerobic and Anaerobic Conversion of Methane into Methanol. *Angew. Chem. Int. Ed.* **2018,** *57* (29), 8906-8910.

7. Markovits, M. A. C.; Jentys, A.; Tromp, M.; Sanchez-Sanchez, M.; Lercher, J. A., Effect of Location and Distribution of Al Sites in ZSM-5 on the Formation of Cu-Oxo Clusters Active for Direct Conversion of Methane to Methanol. *Top. Catal.* **2016,** *59* (17), 1554-1563.

8. Pappas, D. K.; Borfecchia, E.; Dyballa, M.; Pankin, I. A.; Lomachenko, K. A.; Martini, A.; Signorile, M.; Teketel, S.; Arstad, B.; Berlier, G.; Lamberti, C.; Bordiga, S.; Olsbye, U.; Lillerud, K. P.; Svelle, S.; Beato, P., Methane to Methanol: Structure–Activity Relationships for Cu-CHA. *J. Am. Chem. Soc.* **2017,** *139* (42), 14961-14975.

9. Knorpp, A. J.; Pinar, A. B.; Newton, M. A.; Sushkevich, V. L.; van Bokhoven, J. A., Copper-Exchanged Omega (MAZ) Zeolite: Copper-concentration Dependent Active Sites and its Unprecedented Methane to Methanol Conversion. *ChemCatChem* **2018,** *10* (24), 5593-5596.

10. Wieser, J.; Wardecki, D.; Fischer, J. W. A.; Newton, M. A.; Dejoie, C.; Knorpp, A. J.;

Hansen, T. C.; Jeschke, G.; Rzepka, P.; van Bokhoven, J. A., Quantifying the Hydration-Dependent Dynamics of Cu Migration and Activity in Zeolite Omega for the Partial Oxidation of Methane. *Angew. Chem. Int. Ed.* **2024,** *63* (49), e202407395.
